# Reductive Metabolism Influences the Toxicity and Pharmacokinetics of the Hypoxia-Targeted Benzotriazine Di-Oxide Anticancer Agent SN30000 in Mice

**DOI:** 10.3389/fphar.2017.00531

**Published:** 2017-08-11

**Authors:** Yongchuan Gu, Tony T.-A. Chang, Jingli Wang, Jagdish K. Jaiswal, David Edwards, Noel J. Downes, H. D. Sarath Liyanage, Courtney R. H. Lynch, Frederik B. Pruijn, Anthony J. R. Hickey, Michael P. Hay, William R. Wilson, Kevin O. Hicks

**Affiliations:** ^1^Experimental Therapeutics Group, Auckland Cancer Society Research Centre, School of Medical Sciences, The University of Auckland Auckland, New Zealand; ^2^Cancer Research Centre for Drug Development, Cancer Research UK (CRUK) London, United Kingdom; ^3^Sequani Ltd. Ledbury, United Kingdom; ^4^School of Biological Sciences, The University of Auckland Auckland, New Zealand

**Keywords:** bioreductive prodrugs, tumor hypoxia, benzotriazine *N*-oxides, SN30000, tirapazamine, reductive metabolism, hypothermia, pharmacokinetic models

## Abstract

3-(3-Morpholinopropyl)-7,8-dihydro-6*H*-indeno[5,6-*e*][1,2,4]triazine 1,4-dioxide (SN30- 000), an analog of the well-studied bioreductive prodrug tirapazamine (TPZ), has improved activity against hypoxic cells in tumor xenografts. However, little is known about its biotransformation in normal tissues. Here, we evaluate implications of biotransformation of SN30000 for its toxicokinetics in NIH-III mice. The metabolite profile demonstrated reduction to the 1-*N*-oxide (M14), oxidation of the morpholine side-chain (predominantly to the alkanoic acid M18) and chromophore, and subsequent glucuronidation. Plasma pharmacokinetics of SN30000 and its reduced metabolites was unaffected by the presence of HT29 tumor xenografts, indicating extensive reduction in normal tissues. This bioreductive metabolism, as modeled by hepatic S9 preparations, was strongly inhibited by oxygen indicating that it proceeds via the one-electron (radical) intermediate previously implicated in induction of DNA double strand breaks and cytotoxicity by SN30000. Plasma pharmacokinetics of SN30000 and M14 (but not M18) corresponded closely to the timing of reversible acute clinical signs (reduced mobility) and marked hypothermia (rectal temperature drop of ∼8°C at nadir following the maximum tolerated dose). Similar acute toxicity was elicited by dosing with TPZ or M14, although M14 did not induce the kidney and lung histopathology caused by SN30000. M14 also lacked antiproliferative potency in hypoxic cell cultures. In addition M14 showed much slower redox cycling than SN30000 in oxic cultures. Thus a non-bioreductive mechanism, mediated through M14, appears to be responsible for the acute toxicity of SN30000 while late toxicities are consistent with DNA damage resulting from its one-electron reduction. A two-compartment pharmacokinetic model, in which clearance of SN30000 is determined by temperature-dependent bioreductive metabolism to M14, was shown to describe the non-linear PK of SN30000 in mice. This study demonstrates the importance of non-tumor bioreductive metabolism in the toxicology and pharmacokinetics of benzotriazine di-oxides designed to target tumor hypoxia.

## Introduction

The inefficient microvascular system in many human tumors results in regions of hypoxia, which represents a potential tumor-selective target that can be exploited by prodrugs that are activated by metabolic reduction in the absence of oxygen (Wilson and Hay, 2011; Phillips, 2016). This hypoxia-activated prodrug (HAP) strategy not only exploits tumor hypoxia but also targets a subpopulation of tumor cells that is resistant to many anticancer agents (Tredan et al., 2007). The evidence that hypoxic cells limit outcomes with standard-of-care therapy is strongest for radiotherapy of head and neck squamous cell carcinomas (HNSCC), where the extent of hypoxia predicts tumor recurrence (Nordsmark et al., 2005). In addition, the oxygen-mimetic radiosensitizer nimorazole improves outcome only in patients with tumors classified as hypoxic on the basis of gene expression signature (Toustrup et al., 2011). The most extensively studied HAP, the benzotriazine di-oxide tirapazamine (TPZ) showed promising activity in a phase II trial in combination with chemoradiation for HNSCC (Rischin et al., 2005), but failed to achieve its primary endpoint of improved overall survival in a pivotal phase III (Rischin et al., 2010). However, the latter trial was compromised by poor radiotherapy quality control at some centers (Peters et al., 2010) and, likely, by failure to select for patients with hypoxic tumors (Rischin et al., 2006; Trinkaus et al., 2014). Thus, the question as to whether HAPs can improve outcomes in radiation therapy remains open.

**FIGURE 1:**
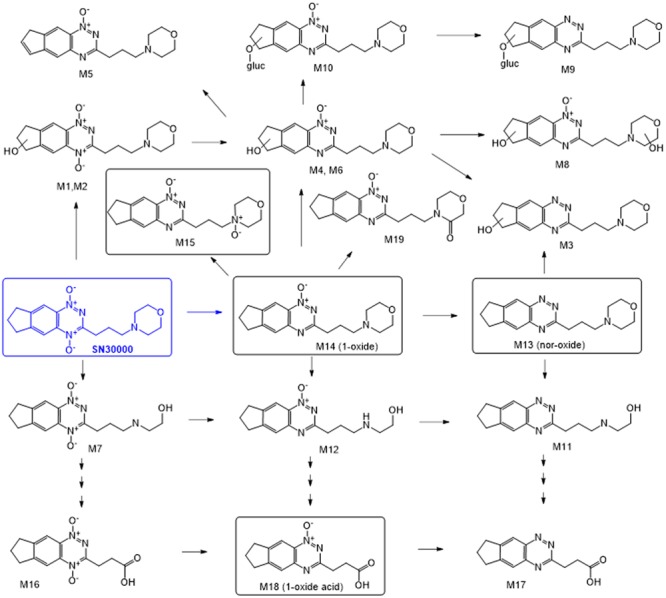
Murine metabolites of the benzotriazine di-oxide SN30000. Structures of metabolites confirmed by synthesis are shown in boxes. Others are inferred from mass spectrometry and absorbance spectra (see **Table 1** and Supplementary Figure 2).

Evidence that the activity of TPZ against hypoxic cells in tumors is limited by inadequate penetration from blood vessels (Durand and Olive, 1997; Hicks et al., 1998, 2003, 2006; Kyle and Minchinton, 1999) led us to develop an analog (SN30000, previously also known as CEN-209; see **Figure 1**) with improved tissue diffusion properties and greater therapeutic activity than TPZ in preclinical models (Hicks et al., 2010; Hay et al., 2017). These studies included a preliminary analysis of plasma PK in CD-1 nude mice and Sprague-Dawley rats, showing higher AUC than for TPZ at equivalent host toxicity, and demonstrated similar histopathology for both agents at or above their maximum tolerated dose (MTD). Analogous to TPZ (Laderoute and Rauth, 1986; Baker et al., 1988), metabolism of SN30000 in hypoxic cell cultures was shown to generate two-electron and four-electron reduced metabolites (the 1-*N-*oxide and nor-oxide, respectively; M14 and M13 in **Figure 1**) which lacked cytotoxicity in culture (Hicks et al., 2010). This is consistent with the critical role of the initial one-electron reduced radical as an intermediate in the hypoxic cytotoxicity of TPZ (Laderoute et al., 1988); this initial reducing radical decays spontaneously to a DNA-reactive oxidizing radical (Daniels and Gates, 1996; Shinde et al., 2009) as subsequently also demonstrated for SN30000 (Anderson et al., 2014). The 1-oxide and nor-oxide species were also identified as major metabolites of SN30000 in liver and HCT116 tumor xenografts in NIH-III mice (Wang et al., 2012), suggesting the major biotransformation route in mice is reduction of the *N*-oxide moieties as is the case for TPZ (Walton and Workman, 1993). A further study demonstrated photodegradation of SN30000 in solution via transfer of the 4-*N*-oxygen to the side chain to generate a morpholino-*N*-oxide with low toxicity both in cell culture and in mice (Gu et al., 2014).

These studies demonstrated that SN30000 induces an acute toxicity in mice, characterized by immobility and coldness to touch, as well as later histological changes (Hicks et al., 2010; Gu et al., 2014). The rapidity of onset of the acute signs (within minutes) would seem inconsistent with DNA damage as the cause, leading to the hypothesis that there is a second mechanism of normal tissue toxicity. This led us, in the present study, to undertake a full description of the biotransformation of SN30000 in mice, and to investigate the relationships between its metabolism, pharmacokinetics and toxicity. We also address a number of issues relevant to the clinical development of SN30000 including dose dependence of PK, whether metabolism within a hypoxic tumor significantly modifies whole body clearance and circulating metabolite concentrations, and whether the prodrug crosses the blood–brain barrier. The examination of brain PK is of particular significance given the increasing importance of brain metastases and primary brain tumors as limiting treatment outcomes as a result of ongoing advances in the management of non-CNS malignancies. In addition, there is compelling evidence for hypoxia in brain tumors and for its role in tumor progression and resistance to therapy (Amberger-Murphy, 2009; Murat et al., 2009; Beppu et al., 2015), making it important to identify HAPs able to access such tumors even in regions in which the blood-brain barrier is not compromised.

## Materials and Methods

### Chemicals

SN30000 and metabolites M14 and M13 (Hicks et al., 2010) and photodegradation product M15 (Gu et al., 2014) were synthesized and formulated as the hydrochloride salts, as were octadeuterated stable isotope internal standards of SN30000, M14 and M13 (Wang et al., 2012). The synthesis and characterization of metabolite M18 is provided in Supplementary Data. ^3^H-mannitol (0.74 TBq/mmol) was from American Radiolabeled Chemicals (St. Louis, MO, United States). Acetonitrile (MeCN; LC-MS grade) was from Merck (Whitehouse Station, NJ, United States) and all other reagents were of analytical grade.

### Animals and Tumor Model

All animals were housed at 19–23°C with a 12 h light/dark cycle. NIH-III nude mice (Crl:NIH-Lystbg Foxn1nu Btkxid) were bred by the Animal Resources Unit (University of Auckland, New Zealand), housed in microisolator cages (Techniplast, Buguggiate, Italy) and fed a sterilized rodent diet (Harlan Teklad diet, 2018i). Tumors were grown subcutaneously on the right flank by inoculation of 10^7^ HT29 cells in 100 μL αMEM culture medium. Mice were dosed when tumors reached approximately 10 mm mean diameter. All animal studies were approved by the University of Auckland Animal Ethics Committee (approvals AEC R830 and CR1190).

### Formulation, Dosing, Clinical Observations, and Sample Collection

SN30000, M14 and M18 dosing solutions were prepared fresh in normal saline, filtered through 0.45 μm Acrodisc filters (Pall Life Sciences, Port Washington, NY, United States), protected from light in amber vials, and used within 3 h. Doses are expressed as mg of free base equivalent for SN30000 and M14, and free acid in the case of M18. Mice (20–25 g body weight) were dosed i.p. or by slow bolus (10–20 s) i.v. injection via a lateral tail vein, with co-administration of ^3^H-mannitol (0.025 μmol/kg; 67 MBq/g) in some studies as a marker of renal function. For evaluation of acute toxicity, mice were observed continuously for 3 h after dosing, and their activity rated on a lethargy score from 0 representing no change in activity to 5 representing animals that were moribund and required culling. For MTD studies, the animals were checked daily for clinical signs of toxicity and body weight change for 2 weeks. In some studies rectal temperature changes after dosing were measured using a BAT-12 digital microprobe thermometer (Physitemp Instruments, Clifton, NJ, United States) at the times specified in the figures. Blood samples were collected from mice at various times by cardiac puncture, seconds after cervical dislocation, into ice-cold tubes containing K_2_EDTA. These were placed on ice immediately after collection and centrifuged (3,000 *g*, 3 min) within 10 min to harvest plasma. Plasma and tissues were rapidly frozen in liquid nitrogen before storage at -80°C for subsequent bioanalysis. Urine samples were collected from mice housed individually in metabolic cages (Minor Metabowl, Jencons, United Kingdom; one animal per cage) with continuous access to food and water.

### Metabolite Profiling

Mouse plasma and tissue homogenates, prepared from tissue samples in two volumes of ice-cold PBS using a TissueLyser II (Qiagen Sciences, Germantown, MD, United States) with 30 Hz oscillation frequency for 1 min, were processed by precipitating proteins with 3 vol MeOH and centrifuging (13,000 *g*, 4°C for 10 min). Supernatants were diluted into 3 vol water and analyzed with an Agilent model 6460 LC-MS/MS with an in-line photodiode array absorbance detector. Chromatographic separation was performed on a Zorbax Eclipse C18 column (100 mm × 3.0 mm, 1.8 μm; Agilent Technologies) at 35°C with a flow rate of 0.6 mL/min, with positive or negative mode electrospray ionization. The mobile phase was a gradient constructed using 80% MeCN/20% water/0.1% formic acid v/v/v (A) and 0.1% formic acid in water (B) from 10% A to 80% A with a total run time of 19.5 min. The gradient profile was: 0–4 min, 10% A; 4–11 min linear increase to 60% A; 11–12 min to 80% A; held for 1 min, returned to 10% A over 0.5 min and held for 2 min before the next sample injection. Absorbance spectra were collected from 230 to 600 nm with a reference wavelength of 550 nm. The mass/charge (*m/z*) ratio was scanned from 100 to 800 with a fragmentor voltage of 120 V. MS^2^ with various collision energies was used for further characterization of some metabolites.

### Redox Cycling of SN30000 and M14

The Chinese hamster ovary cell line 51D1.3 (herein called CHO) and a stable transfectant expressing a soluble form of human P450 oxidoreductase (51D1.3/sPOR, herein called CHO/POR) (Gu et al., 2009), were grown in spinner flasks in αMEM plus 5% fetal bovine serum. Late log phase cultures were harvested by centrifugation, resuspended at 5 × 10^5^ cells/mL in phenol red-free high-glucose DMEM (Invitrogen) supplemented with 4 mM Glutamax-1, 1 mM sodium pyruvate and 5% FBS. Stirred cell suspensions (2 mL) were transferred to an OXYBOROS O2K oxygraph for simultaneous polarographic measurement of non-respiratory O_2_ consumption and fluorimetric measurement of H_2_O_2_ generation as previously (Hunter et al., 2012). Bovine liver superoxide dismutase (10 U), horseradish peroxidase (10 U), Amplex UltraRed (50 nmol) and rotenone (1 nmol), all from Molecular Probes Invitrogen (Eugene, OR), were injected and cumulative drug additions (from saline stock solutions) were made every ∼5 min once endogenous rates had stabilized. Signals were recorded every 2 s, and flux derivatives were estimated ∼2 min after each addition using a Savitzky–Golay smoothing filter (40 data point window) in DATLAB version 4.3.

### S9 Metabolism of SN30000 and M14

*In vitro* hepatic metabolism of SN30000 and M14 was studied using 9,000 *g* post-mitochondrial supernatants (S9) prepared from livers of female NIH-III mice. Metabolism at 37°C was measured by dilution of S9 to appropriate protein concentrations (measured by BCA protein assay) in sodium/potassium phosphate buffer (67 mM phosphate, pH 7.4) containing 5 mM MgCl_2_, 1 mM EDTA, 1 mM NADPH, 1 mM NADH and 150 μM test drug. The reaction was terminated by adding an equal volume of ice-cold MeOH and samples were analyzed by LC-MS/MS. Two methods were used to modify the oxygen concentration. Method 1: 96-well plates (reaction volume 0.1 mL/well) were incubated for 30 min on a Eppendorf Thermomixer C (Eppendorf AG, Hamburg, Germany) either under 21% O_2_ (oxia) or 95% N_2_/5% CO_2_ (anoxia) in an anaerobic chamber (Sheldon Manufacturing, Cornelius, OR, United States) as described previously (Gu et al., 2010). Method 2: glass vials containing 3 mL final volume were magnetically stirred continuously in a water bath under flowing 5% CO_2_ containing 0, 0.2, 2, or 20% O_2_ (certified gas mixtures from BOC Gasses NZ), as detailed previously (Hicks et al., 2007), and samples (0.1 mL) were withdrawn at intervals for analysis.

### Bioanalysis of SN30000 and Its Metabolites

Mouse PK and S9 metabolism samples were analyzed using an LC-MS/MS method adapted from a previously reported method (Wang et al., 2012), with octadeuterated internal standards for SN30000, M14 and M13 (see Supplementary Data). M18 was quantified by absorbance detection with reference to an external standard calibration curve. The analytical platform was as for the metabolite profiling above, except that the column was a Zorbax SB-C18 (1.8 μm, 50 mm × 2.1 mm; Agilent Technologies), the flow rate was 0.5 mL/min and the gradient profile (6 min run time) was: 0–1 min, 10% A; 1–4 min, 10–60% A; 4–5 min, 60 to 80% A, and 5–5.5 min, 80 to 10% A. Electrospray ionization was used in positive mode, with source parameters and MS transitions as previously (Wang et al., 2012). Validation of the method for the concentration ranges 0.01–50 μM (SN30000), 0.005–25 μM (M14) and 0.002–10 μM (M13) is reported in Supplementary Tables 1–4.

### Non-compartmental and Compartmental Modeling of Pharmacokinetics

Concentration-time data were analyzed by non-compartmental analysis using Phoenix WinNonlin (ver 6.4, Build 6.4.0768 Pharsight, United States); all AUC values were extrapolated to infinity using the linear trapezoidal rule. A compartmental model for plasma PK after dosing with SN30000 or M14 was fitted using Phoenix NLME v1.3 with random effects on the metabolite volumes of distribution, and temperature effects based on the observed body temperature at the last measurement time. Temperature effects were added or removed until there was no significant change in the log likelihood. Model fits were further assessed by visual inspection of the residuals and predicted PK.

### Statistical Methods

Linear and non-linear regressions were performed using Sigmaplot 13.0. Data and parameters from regressions are presented as means and SEM with the number of independent determinations indicated. Comparison of means was performed by two tailed *t*-tests using Sigmaplot 13.0.

## Results

### SN30000 Metabolite Profile in Mice

Metabolites were investigated in plasma, liver and HT29 tumors following i.p. dosing of female NIH-III mice with SN30000 at 186 mg/kg, which represents its previously determined MTD for this route and mouse strain (Gu et al., 2014). Representative chromatograms are shown in Supplementary Figure 1. A total of 19 metabolites (M1–M19) were provisionally identified (**Figure 1** and **Table 1**). Assignments were confirmed by comparison with synthetic standards in the case of the 1-oxide M14 and nor-oxide M13, which have previously been reported as murine metabolites of SN30000 (Wang et al., 2012) and for M15 which is known to be the major photodegradation product of SN30000 (Gu et al., 2014). In addition, the previously unreported metabolite M18, which is the main plasma metabolite at late times, was isolated from pooled plasma of mice dosed with M14 in sufficient quantity for characterization by NMR (Supplementary Data). This identified M18 as an oxidation product of M14 with an alkanoic acid side-chain (**Figure 1**), an assignment that was confirmed by synthesis of the authentic compound which was identical by HPLC retention time, absorbance spectrum, elemental analysis, mass spectrum and NMR spectroscopy.

**Table 1:** Metabolites tentatively identified in NIH-III mice after administration of a single dose of SN30000.

			Mass spectrum (*m/z*)		Metabolite/SN30000 peak area^c^		
ID	RRT^a^	λ_max_^b^	Parent	Product ions	Plasma	Liver	Tumor
M1	0.23	252,300,400	347	100,133,228,260	0.18	ND^d^	0.19
M2	0.31	252,300,400	347	100, 228,260	0.03	ND	0.02
M3	0.51	245,325	315	100,210,228	0.07	0.55	0.12
M4	0.56	245,360	331	100,227,244	0.22	0.83	0.35
M5	0.67	255,345	313	100,156,226	0.05	0.64	0.04
M6	0.79	252,370	331	227,244	0.10	0.35	0.13
M7	0.89	252,300,400	305	117,198,214,244	0.06	0.17	0.06
M8	0.92	252,360	347	227,244	0.05	ND	0.04
M9	0.95	252,330	491	100,228,315	0.04	0.36	0.08
SN30000	1.00	252,300,400	331	100,212,244	1.00	1.00	1.00
M10	1.06	260,360	507	244,331	0.31	0.47	0.44
M11	1.15	252,320	273	212	0.04	0.34	0.06
M12	1.22	252,360	289	211,228	0.13	1.06	0.16
M13	1.23	245,325	289	100,212	0.08	1.50	0.11
M14	1.29	252,360	315	211,228	0.35	5.18	0.41
M15	1.38	252,360	331	211,228	0.26	0.21	0.24
M16	1.39	252,300,400	276	130,214,230,258	0.50	0.34	0.49
M17	1.67	245,320	244	142,168,198,226	0.11	0.12	0.14
M18	1.73	252,360	260	130,172,214,242	1.14	0.60	1.63
M19	1.81	252,360	329	112,172,200,228,311	0.03	0.27	0.02

*For proposed structures, see **Figure 1**.*

*^a^Retention time relative to SN30000.*

*^b^Absorbance maxima (nm), determined with an HPLC photodiode array detector.*

*^c^Values are peak area ratios for representative animals based on absorbance detection at 252 nm, 30 min after i.p. dosing with SN30000 at 186 mg/kg.*

*^d^ND = Not detected by absorbance or MS.*

Additional structural assignments were based on mass and absorbance spectra, the latter utilizing a characteristic shift in wavelength of the absorbance maximum (λ_max_) on reduction of the benzotriazine di-oxide chromophore to the 1-oxide and nor-oxide (Gu et al., 2014). Absorbance spectra and precursor and product ion mass spectra are provided in Supplementary Figure 2. To characterize the structures of the metabolites, we first studied the product ion spectrum derived from the [M+H]^+^ parent molecular ion of SN30000 (*m/z* 331). Collision-induced dissociation resulted in product ions at *m/z* 244 and 212 corresponding to two 3-alkylbenzotriazine fragments, and product ions of the morpholine moiety at *m/z* 114 and 100. These characteristic product ions were subsequently used to assist with interpretation of metabolite structures as detailed in Supplementary Figure 2. For example, metabolite M19 showed its [M+H]^+^ ion at *m/z* 329, 2 units lower than that of SN30000. The product ions at *m/z* 112 and 98 for M19 were also 2 units lower than the morpholine ring product ion for SN30000, whereas the other two product ions were identical (*m/z* 228, 200). Hence oxidation of the morpholine ring of M14 appears to be the only structural modification in M19.

### *In Vitro* Cytotoxicity and Mouse Toxicity of the Major Metabolites M14 and M18

The prominent 1-oxide (M14) and nor-oxide (M13) metabolites of SN30000 are less potent than SN30000 in antiproliferative assays, especially under hypoxic conditions (Hicks et al., 2010; Gu et al., 2014). We found the 1-oxide alkanoic acid M18 to be even less cytotoxic than the initial 1-oxide metabolite M14, and that it is at least 1000-fold less potent than SN30000 itself in anoxic HCT116 cultures (Supplementary Table 5). This suggested the 1-oxide metabolites are unlikely to be responsible for mouse toxicities that result from antiproliferative effects of SN30000. However, as previously noted (Gu et al., 2014) SN30000 causes an acute toxicity characterized by immobility and coldness to touch which develops within minutes of dosing and resolves after several hours. We therefore compared the mouse toxicity of SN30000 and M14 following single i.p. doses by evaluating clinical signs, body weight loss and survival at 14 days (Supplementary Table 6). This established that the MTD of SN30000 and M14 were the same on a molar basis (562 μmol/kg, equivalent to 186 mg/kg for SN30000 or 177 mg/kg for M14) and that M14 caused similar acute clinical signs to SN30000 (cold skin, lethargy and shivering, progressing to a variable duration of almost complete inactivity for 1–2 h but with no loss of consciousness). The acute effects of M14 seemed slightly more severe than SN30000, although it caused less subsequent body weight loss than SN30000 (Supplementary Table 6). Histopathology of major tissues 2 weeks after dosing confirmed that the toxicology of the two compounds is qualitatively different. As detailed in Supplementary Table 7, pathology findings following SN30000 were most marked for kidney with dose-dependent abnormalities including basophilic staining of the cortical tubules, karyomegaly in the tubular epithelium and mild to moderate tubular epithelial necrosis. In contrast, no histopathological changes were found following dosing with M14 (Supplementary Table 7).

### SN30000 Pharmacokinetics in Mice with and without HT29 Tumors

We then investigated PK of SN30000 and its major metabolites in plasma and tissues after dosing mice i.p. at the MTD of 186 mg/kg. Since hypoxia in tumors could potentially cause increased clearance by bioreduction, we compared the PK of SN30000 in both tumor-free mice and mice bearing HT29 human colon carcinomas that constituted approximately 2% of body weight. To minimize post-mortem drug metabolism (demonstrated to occur rapidly in liver, for SN30000 but not M14, if tissue processing was delayed; Supplementary Figures 6, 7), mice were culled by cervical dislocation, a small blood sample was rapidly collected by cardiac puncture after opening the abdomen, the liver was sampled and frozen within 3 min and tumor or brain (from non-tumor bearing mice) was collected last. SN30000, M14 and M13 were quantified by LC-MS/MS using stable isotope internal standards, while M18 was quantified by absorbance detection using external standard calibration curves.

**FIGURE 2:**
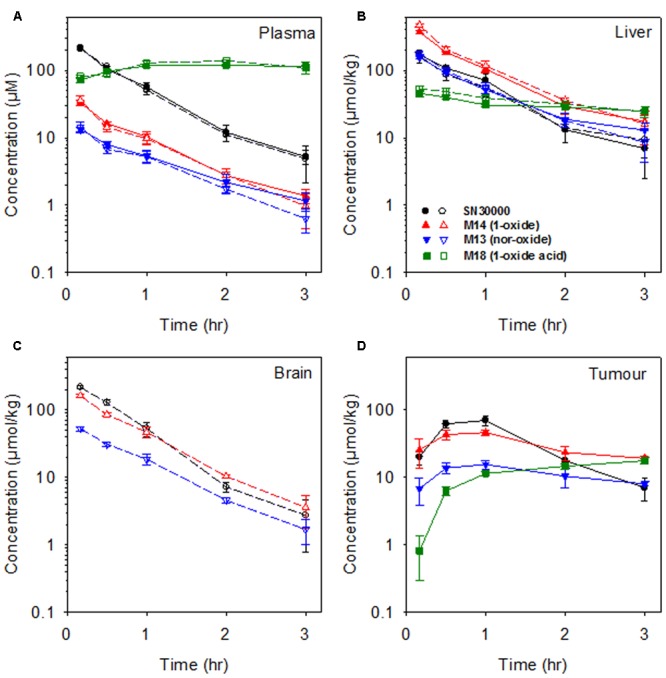
Concentrations of SN30000 and its 1-oxide (M14), nor-oxide (M13) and 1-oxide acid (M18) metabolites in **(A)** plasma, **(B)** liver, **(C)** brain, and **(D)** HT29 tumor xenografts after i.p. administration of SN30000 at 186 mg/kg to female NIH-III nude mice. Filled symbols and solid lines: tumor-bearing mice. Open symbols and dashed lines: non-tumor-bearing mice. Values are means and errors are SEM for ≥3 animals at each time point.

Concentration-time curves for SN30000, M14, M13, and M18 are shown in **Figure 2** and non-compartmental PK parameters in Supplementary Table 8. The presence of HT29 tumor xenografts had no effect on the PK of SN30000 or its metabolites. Maximal concentrations of SN30000, M14 and M13 were all reached within 5 min after i.p. dosing, followed by approximately mono-exponential elimination with a half-life of ∼30 min for each compound in all tissues except for a marked lag in the PK in tumors. Notably, concentrations of SN30000 in brain were similar to plasma, and M14 and M13 concentrations were higher in brain than plasma. In contrast, the alkanoic acid M18 was not detected in brain, but accumulated to high concentrations (>100 μM) in plasma where it was the major metabolite by 1 h with negligible elimination up to 3 h.

**FIGURE 3:**
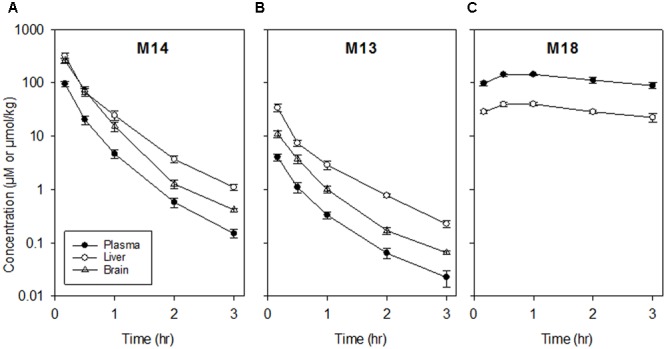
Pharmacokinetics of M14 and its major metabolites in plasma, liver and brain following dosing of female NIH-III mice with M14 (135 mg/kg, i.p.). Values are means and SEM for four animals. **(A)** M14. **(B)** M13. **(C)** M18 (not detectable in brain).

When mice were dosed with authentic M14 (135 mg/kg, i.p.), concentrations of M14 and M13 were higher in brain (and liver) than plasma, while M18 was a major metabolite in plasma and liver but was again undetectable in brain (**Figure 3**; non-compartmental PK parameters are reported in Supplementary Table 9). Thus the high concentrations of M14 and M13 in brain reflect partitioning from plasma rather than bioreductive metabolism in the brain.

### Effects of SN30000, M14 and M18 on Core Body Temperature in Mice

**FIGURE 4:**
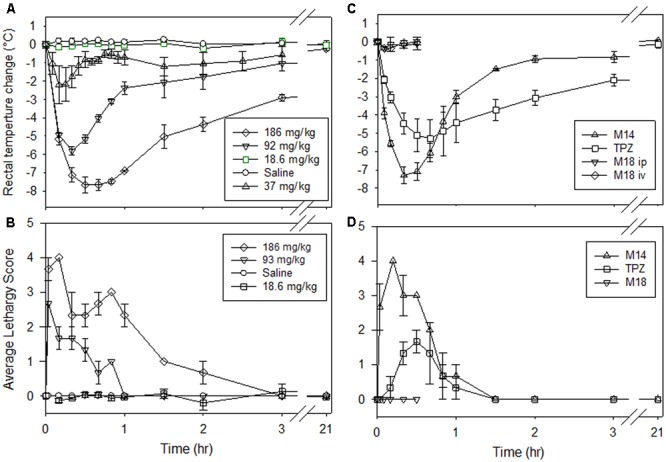
Changes in mouse body temperature and activity after dosing of NIH-III mice. **(A,B)** i.p. saline (vehicle) or SN30000 at the indicated doses. **(C,D)** TPZ (i.p., 31.7 mg/kg) or M14 (i.p., 135 mg/kg, equimolar with SN30000 at 186 mg/kg) at their respective MTDs, or M18 (36 mg/kg i.p. or i.v.). **(A,C)** Mouse rectal temperature as change from baseline (36.6 ± 0.1°C for all animals). **(B,D)** Activity level as a lethargy score from 0 (before treatment) to 5 (moribund and requiring culling). Values are mean and SEM for three animals.

The drop in body temperature, noted above after dosing with SN30000 or M14, led us to explore the impact of this drug-induced hypothermia on the PK and metabolism of SN30000. As a first step, we quantified the core temperature changes using a rectal probe (**Figure 4**). SN30000 induced a dose-dependent drop in temperature, with a nadir ∼8°C below controls at approximately 30 min following an MTD dose (**Figure 4A**). The initial rate of temperature decline at this dose was almost as rapid as the post-mortem temperature drop in non-drug-treated animals (Supplementary Figure 3). Physical activity of the animals, evaluated using a semi-quantitative lethargy score, approximately tracked the temperature drop (**Figure 4B**). A similar drop in core body temperature (**Figure 4C**) and mobility (**Figure 4D**) was observed following dosing with M14 at its MTD, with slightly faster recovery than after SN30000. TPZ also induced hypothermia (**Figure 4C**), again with a time course similar to that for increased lethargy (**Figure 4D**) following dosing at its MTD (31.7 mg/kg). In contrast, when M18 was administered i.p. or i.v. at 36 mg/kg, resulting in plasma concentrations ca. three–fourfold higher than after SN30000 administration at its MTD (data not shown), no acute clinical signs or hypothermic response were observed (**Figures 4C,D**). The MTD of M18 has not been determined but no body weight loss was observed at this dose level (Supplementary Table 6). Stimulation of muscaric acetylcholine receptors through acetylcholinesterase (AChE) inhibition commonly induces hypothermia in rodents (Gordon et al., 2014). We tested whether these compounds inhibit mouse brain acetylcholinesterase activity; both SN30000 and M14 were low potency inhibitors (IC_50_ values of 131 and 58 μM, respectively), whereas M18 and TPZ showed no inhibition at 300 μM (Supplementary Table 10). However, predosing of the mice with atropine had no effect on SN30000-induced hypothermia (data not shown).

### Comparison of Redox Cycling of SN30000 and M14

**FIGURE 5:**
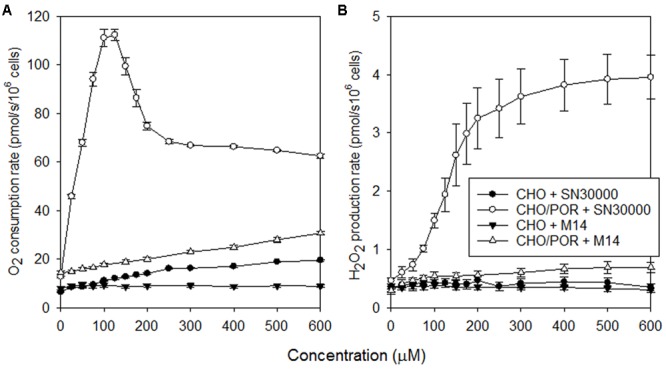
Rates of non-respiratory O_2_ consumption **(A)** and H_2_O_2_ generation **(B)** in rotenone-inhibited stirred aerobic CHO and CHO/POR cell suspensions (5 × 10^5^ cells/ml) during cumulative addition of SN30000 or M14. Values are means and SEM for four independent titrations for each cell line.

Generation of reactive oxygen species via redox cycling in the presence of O_2_ has been implicated in the aerobic cytotoxicity of TPZ and SN30000 (Silva and O’Brien, 1993; Elwell et al., 1997; Hunter et al., 2012), although its contribution to clinical toxicity of TPZ is unclear. The surprising finding that M14 induces similar acute effects to SN30000 in mice led us to evaluate whether M14 is also a substrate for one-electron redox cycling. Non-respiratory (rotenone-resistant) oxygen consumption (**Figure 5A**) and H_2_O_2_ generation (**Figure 5B**) was tested in CHO/POR cells which highly express the human P450 oxidoreductase POR, the main enzyme responsible for one-electron reduction of SN30000 (Hunter et al., 2015). SN30000 strongly stimulated O_2_ consumption and H_2_O_2_ formation as previously reported (Hunter et al., 2012). (Additional studies, reported in Supplementary Figure 4, showed that the decrease in O_2_ consumption rates at >125 μM SN30000 is due to time-dependent decreases in redox cycling, likely reflecting rate-limiting NADPH regeneration, and is not seen when SN30000 is added as single rather than cumulative injections). As also shown in **Figure 5**, redox cycling was induced by M14 but at much lower rates than for SN30000. Investigation of human tumor cells (SiHa and SiHa/POR) using a Seahorse^TM^ flux analyser confirmed stimulation of oxygen consumption by SN30000, and to a lesser extent by TPZ, both with and without inhibition of Complex I by rotenone, while no such stimulation was observed with M14 (Supplementary Figure 5). We conclude that redox cycling of M14 is much less facile than for SN30000 and is therefore unlikely to be responsible for the hypothermia and acute signs induced by these agents.

### Hypoxia and Temperature Dependence of SN30000 Metabolism in S9 Preparations

**FIGURE 6:**
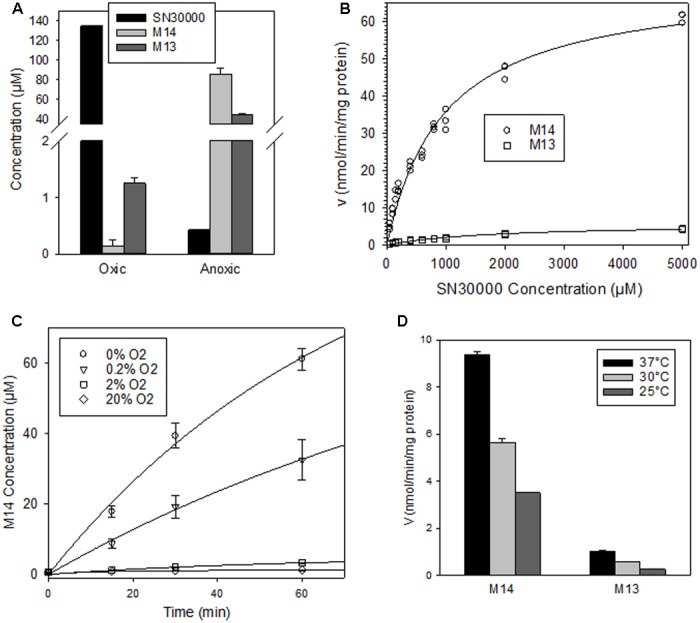
Metabolism of SN30000 in liver S9 preparations from female NIH-III mice. All values are means ± SEM for three replicates with an initial SN30000 concentration of 150 μM and 30 min incubation at 37°C unless otherwise indicated. **(A)** Reduction of SN30000 to 1-oxide M14 and nor-oxide M13 under aerobic (20% O_2_) and anoxic conditions (2 mg/mL protein). **(B)** Kinetics of reduction of SN30000 to M14 and M13 under anoxia (100 μg/mL protein), fitted assuming Michaelis–Menten kinetics. **(C)** Oxygen dependence of inhibition of formation of M14 (100 μg/mL protein). The values in the key are gas phase O_2_ concentrations. **(D)** Temperature dependence of rate of reduction of SN30000 to M14 and M13 under anoxia (100 μg/mL protein).

The above metabolite profiles demonstrated extensive reduction of the 4-*N*-oxide moiety of SN30000 in tumor-free mice. To address whether this reflects an oxygen-sensitive (one-electron reduction) or oxygen-insensitive (two-electron reduction) process, metabolism was compared in NIH-III mouse liver S9 preparations under 20% O_2_ or anoxia. Oxygen dramatically suppressed metabolism of SN30000 to M14 and M13 in mouse liver S9 (**Figure 6A**). Using low protein concentrations (20 μg/ml) to enable assessment of initial rates, reduction to M14 was similar for anoxic liver S9 preparations from male and female NIH-III mice (10.6 ± 0.2 and 9.2 ± 0.2 nmol/min/mg protein, respectively). The SN30000 concentration dependence of formation of M14 and M13 gave high apparent *K*_m_ values of 974 ± 71 μM (*V*_max_ 71 ± 2 nmol/min/mg protein) for formation of M14 with low rates of formation of M13 (**Figure 6B**). The liver S9 intrinsic clearance, CLint, was calculated as *V*_max_/*K*_m_ for M14 formation under anoxia and scaled to the whole liver based on the (unrealistic) assumption that the liver is entirely anoxic. This upper estimate of *in vivo* hepatic clearance was then predicted as previously described (Gu et al., 2011b) using the free fraction of SN30000 in mouse plasma determined by equilibrium dialysis (Supplementary Table 11). The resulting estimates of hepatic clearance were 3.91, 5.01, and 4.69 L/h/kg using well-stirred, parallel tube and dispersion models, respectively; these estimates are comparable to the clearance following i.v. dosing of SN30000 (5–10 L/hr/kg; **Table 2**).

**Table 2:** Non-compartmental plasma PK parameters for SN30000 and its major metabolites after i.v. dosing of female NIH-III nude mice with SN30000 at the indicated doses.

		Dose (mg/kg)		
Compound	Parameter	1.86	18.6	78.3
SN30000	AUC (μmol.hr/L)	0.53	8.10	47.00
	*T*_1/2_ (min)	8.03	27.57	23.94
	*C*_max_ (μmol/L)	1.86 ± 0.13^a^	25.21 ± 0.27	81.94 ± 2.71
	Cl (L/hr/kg)	10.6	6.94	5.04
	*V*_d_ (L/kg)	1.52	1.29	2.24
				
M14	AUC (μmol.hr/L)	0.042	0.60	11.63
	*T*_1/2_ (min)	12.35	28.53	31.52
	*C*_max_ (μmol/kg)	0.129 ± 0.019	1.42 ± 0.19	22.25 ± 2.13
				
M13	AUC (μmol.hr/L)	0.0047	0.146	3.34
	*T*_1/2_ (min)	27.72	47.12	38.93
	*C*_max_ (μmol/kg)	0.0091 ± 0.0013	0.317 ± 0.035	5.84 ± 0.76
				
M18	AUC (μmol.hr/L)	0.77	10.11	60.85
	*T*_1/2_ (min)	–	101.23	74.95
	*C*_max_ (μmol/kg)	0.44 ± 0.04	6.30 ± 1.05	25.25 ± 0.42

*^a^Blood was collected on termination; thus error estimates (SEM, for three mice) are available only for *C*_*max*_.*

We also quantified rates of M14 formation at a range of O_2_ concentrations, using stirred S9 with flowing gas to ensure equilibration (**Figure 6C**). Initial rates of M14 formation were fitted using a 4-parameter Hill model, giving an estimated O_2_ concentration in the gas phase of 0.19 ± 0.03% for 50% inhibition of reductive metabolism of SN30000 to M14. Formation of M14 was temperature dependent, with 40 and 63% inhibition at 30 and 25°C, respectively, relative to 37°C (**Figure 6D**).

### Dose Dependence of SN30000 Pharmacokinetics

**FIGURE 7:**
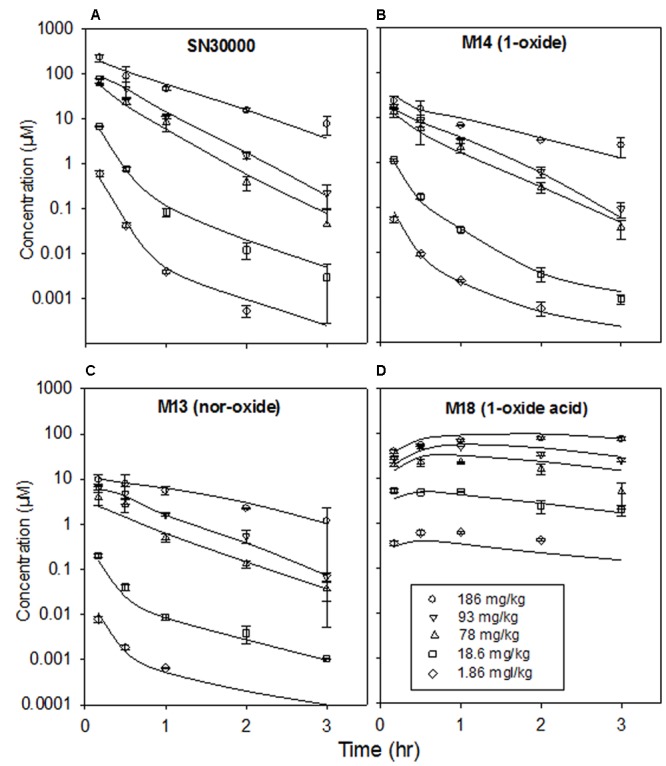
Plasma concentrations of SN30000 and its major metabolites after i.p. administration of SN30000 at the indicated doses to female NIH-III mice. Each point is the mean and SEM for three animals. Lines are fitted using the compartmental PK model described in the text, with clearance parameters dependent on the body temperature changes shown in **Figure 4**.

**FIGURE 8:**
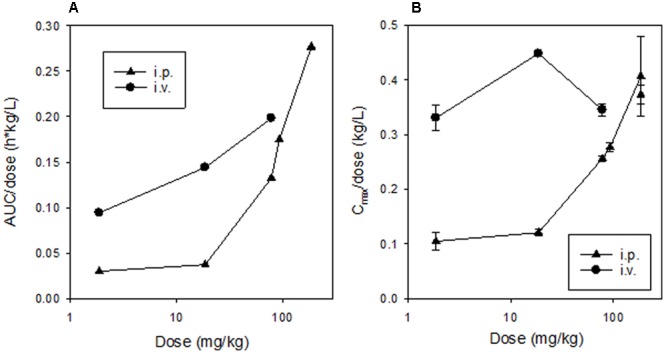
Dose-dependence of non-compartmental pharmacokinetic parameters for SN30000 in plasma following dosing of female NIH-III mice. Values for AUC/dose **(A)** are for groups of three animals (with two datasets for the 186 mg/kg dose) and for *C*_max_/dose **(B)** are means and errors are SEM for ≥3 animals.

Dose-dependence of plasma PK of SN30000 in NIH-III mice was evaluated following i.p. and i.v. administration at five and three dose levels, respectively. Concentration-time profiles for SN30000 and its major metabolites following i.p. dosing showed clear evidence of two-compartment PK at low dose, while the early rapid clearance phase was not clearly resolved at high dose (**Figure 7**). Non-compartmental PK parameters for i.v. and i.p. dosing are listed in **Table 2** and Supplementary Table 12, respectively. The ratios AUC/dose and *C*_max_/dose (**Figure 8**) showed a marked increase at i.p. doses >10% of MTD, while the trend was less pronounced for i.v. dosing over the more limited range imposed by acute toxicity via this route. The i.p. bioavailability of SN30000, based on these AUC estimates, increased with dose from ca 30% at 1–10% of the i.p. MTD to ca. 70% at 42% of the i.p. MTD.

In a separate experiment, urine was collected from female NIH-III mice for 24 h after dosing SN30000 at 18.6 and 186 mg/kg i.p. and analyzed by HPLC; this showed excretion of only 3.38 ± 0.03% and 5.64 ± 0.02% (mean and SEM, *N* = 3), respectively, of the administered dose indicating that urinary excretion is a minor contributor to clearance and cannot account for the non-linear PK of SN30000.

**FIGURE 9:**
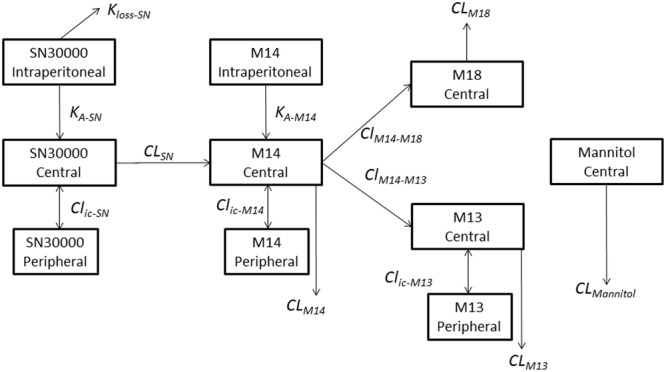
Schematic representation of the compartmental model describing the plasma PK of SN30000, its major metabolites and mannitol in female NIH-III mice. SN30000, M14 and M13 have two compartments (with compound specific volumes of distribution V1 and V2) while M18 and mannitol have one compartment. SN30000 and M14 have additional i.p. compartments for dosing, with respective absorption rate constants, *K*_A_, and a term for first pass metabolism of SN30000 (*K*_Loss_), while i.p. mannitol was assumed to rapidly enter the central compartment. Clearances (CL) and intercompartmental clearances (*Cl*_ic_) are also shown. Parameter values, and their temperature dependencies, are shown in Supplementary Table 13.

To further explore this non-linearity, we developed a two-compartment model to describe plasma PK (with addition of a third, dosing compartment to represent i.p. injection of SN30000 or M14 with absorption rate constants *K*_A_). This model, shown schematically in **Figure 9**, is based on total (free + bound) plasma concentrations; the major metabolites (M14, M13, and M18) are explicit and clearance of SN30000 occurs only by reduction to M14. The i.p. bioavailability of SN30000 was modeled with an additional term for loss from the peritoneal compartment (*k*_loss_). Measured body temperature decreases were assumed to reduce clearance of each compound from the dosing and central compartments to (1+ΔT)^-N^ of its maximal value, where ΔT is the magnitude of the observed temperature drop from baseline and N is compound and compartment specific. Doses ≤18.6 mg/kg were assumed to cause no temperature drop (consistent with **Figure 4A**) while the temperature effect for i.p. dosing with 78.3 mg/kg SN30000 was scaled linearly from the values measured after dosing at 92.8 mg/kg i.p. Model parameters were fitted to all the datasets (SN30000 i.p. and i.v., and M14 i.p.) simultaneously, with ΔT treated as constant during intervals between temperature measurements.

The parameters of the model are listed in Supplementary Table 13. The fitted curves for i.p. dosing with SN30000, shown in **Figure 7**, demonstrate that the model provides a good description of the non-linear PK of SN30000. Similarly good fits were obtained with the other datasets (i.v. SN30000 in Supplementary Figure 8, and i.p. M14 in Supplementary Figure 9). The marked temperature dependence of the central compartment clearance terms, relative to the temperature dependence of S9 metabolism, may indicate other metabolic changes induced by SN30000 dosing.

**FIGURE 10:**
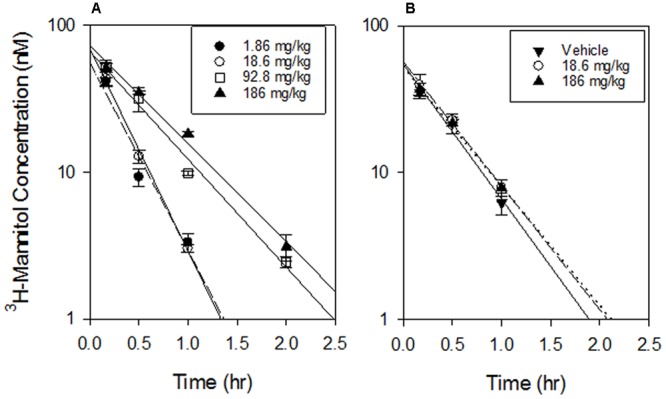
Mannitol PK. **(A)** PK of ^3^H-mannitol co-administered i.p. with the indicated doses of SN30000. Lines are linear regressions of log(mannitol concentration) vs time. There was a significant difference in *T*_1/2_ between the lowest dose and 92.8 mg/kg or 186 mg/kg (*p* < 0.01) but no significant difference between 1.86 and 18.6 mg/kg (*p* = 0.059) or between 92.8 and 186 mg/kg (*p* = 0.5). **(B)** Mannitol PK when administered i.p. 16 h after the indicated i.p. doses of SN30000, showing no significant lasting effect of SN30000 on *T*_1/2_ of mannitol excretion between high dose and vehicle (*p* = 0.57). Values are mean and SEM for three animals.

In experiments where SN30000 was dosed at 1, 10, 50, and 100% of its MTD, ^3^H-mannitol (25 μmol/kg) was co-administered with SN30000 to investigate circulatory or renal disturbances induced by hypothermia. The plasma PK of mannitol changed at high SN30000 doses (**Figure 10**), with a ∼2-fold increase in half-life and 3-fold increase in AUC at the highest i.p. dose of SN30000 (Supplementary Table 14). In contrast, plasma PK of ^3^H-mannitol administered 16 h after i.p. dosing with SN30000 was not significantly affected up to 186 mg/kg SN30000 (Supplementary Table 14) indicating that renal clearance of mannitol had recovered by this time.

## Discussion

Previous studies have reported reductive metabolism of SN30000 to the 1-oxide (M14) and nor-oxide (M13) in mice (Hicks et al., 2010; Wang et al., 2012), analogous to the main route of metabolism of TPZ in rodents and humans (Walton and Workman, 1993; Senan et al., 1997). The more extensive evaluation of metabolite profiles of SN30000 in the present study demonstrates three biotransformation pathways, illustrated in **Figure 1**, namely reduction of the *N*-oxide moieties (initially at the 4-*N*-oxide), oxidation of the morpholine or indane rings, and glucuronidation.

The newly identified 1-oxide acid M18, which is the major plasma metabolite at late times, illustrates both the reductive and oxidative pathways. The resulting propionic acid side chain confers increased plasma protein binding (Supplementary Table 11) which may contribute to its long plasma half-life (**Figure 2**). Dosing of mice with M14 also generated M18, demonstrating reduction followed by oxidation as a route of M18 formation from SN30000 *in vivo.* However, we also observed that the di-oxide acid M16 undergoes facile reduction to M18 in hypoxic mouse liver S9 preparations (data not shown), suggesting that oxidation of SN30000 followed by reduction contributes to M18 formation. M18, like M14, had low cytotoxic potency (Supplementary Table 5), and in contrast to M14 had no observable acute toxicity (**Figure 4**); in addition the time dependence of acute toxicity is inconsistent with M18 PK. Hence M18 is unlikely to be of toxicological significance but as a long-lived metabolite may be a useful biomarker of whole-body reductive metabolism of SN30000.

A surprising finding is that the initial 1-oxide metabolite, M14, induces acute toxicity (including marked hypothermia) similar to that for SN30000 at equimolar doses and may mediate the acute toxicity of SN30000. The initial rate of temperature drop after dosing with SN30000 (or TPZ) is almost as rapid as that measured post-mortem in non-drug-treated mice (Supplementary Figure 3) indicating rapid and almost complete suppression of thermogenesis. To our knowledge this hypothermic response has not been previously reported for TPZ, although similar effects have been described for high doses of nitroimidazole radiosensitizers (Haynes and Inch, 1976; Conroy et al., 1980). The 1-oxide metabolites of benzotriazine di-oxides have previously been considered benign because of their low cytotoxic potency in culture (Baker et al., 1988; Siim et al., 2004; Hicks et al., 2010) as confirmed by Supplementary Table 5. The acute toxicity of M14 is unlikely to be caused by redox cycling given the much lower rates of oxygen consumption and H_2_O_2_ generation by M14 than SN30000 in rotenone-inhibited CHO cells (**Figure 5**), reflecting its 70 mV lower one-electron reduction potential (Anderson et al., 2014). We note that the dramatic temperature drop following dosing with SN30000 and TPZ may be a complicating factor in interpreting preclinical studies in which these agents have been dosed shortly before combination with radiation or other drugs (e.g., Brown and Lemmon, 1990; Dorie et al., 1994; Dorie and Brown, 1997; Hicks et al., 2010). The effect of M14 on body temperature is expected to be less severe in larger animals because of slower passive heat loss, which is supported by our preliminary observations of temperature changes in Wistar rats which showed a decrease in rectal temperature of only ∼2.5°C after a single i.v. dose of 90 mg/kg, which is the MTD in these animals (data not shown). Both SN30000 and M14 inhibited mouse brain homogenate acetylcholinesterase activity in the high micromolar range whereas M18 and TPZ showed no inhibition at 300 μM (Supplementary Table 10) which is similar to reported negative findings for metronidazole and misonidazole (von Burg and Conroy, 1979). However, predosing of the mice with atropine had no effect on SN30000-induced hypothermia (data not shown). There was also no clear effect of SN30000 or M14 on mitochondrial function in a standard Seahorse mitochondrial stress test (Supplementary Figure 5) suggesting that the compounds do not affect oxidative phosphorylation coupling, at least in human cancer cells. Thus the molecular mechanism(s) responsible for the loss of thermogenesis in mice is not clear, but may have broader toxicological implications and warrants further investigation. We also note that clinically significant toxicities of TPZ in humans include fatigue, nausea, vomiting, diarrhea, muscle cramping and tinnitus (Doherty et al., 1994; Johnson et al., 1997; Senan et al., 1997) many of which may be unrelated to antiproliferative effects. Thus there may be opportunity to develop improved BTOs by counter-screening the corresponding 1-oxide metabolites to minimize their acute toxicity.

In contrast to their similar acute toxicity profiles, SN30000 induced multiple histopathological changes in mice whilst M14 did not. The tissue toxicity profile of SN30000 was typical of DNA-reactive cytotoxins (bone marrow, testes, thymus, spleen), but also with marked kidney toxicity manifested as basophilia, karyomegaly, and necrosis of the cortical tubules. We note that the HAP evofosfamide also induces kidney toxicity in rats (Bendell et al., 2009); hypoxia in the normal renal medulla (Haase, 2013) may underlie the sensitivity of this organ to HAPs, although kidney toxicity has not emerged as dose-limiting for this class of agents in humans.

Extensive bioreductive metabolism of SN30000 signaled by the metabolite profile in rodents was confirmed by metabolism in S9 preparations, which was markedly enhanced by hypoxia (**Figure 6**). Initial rates of M14 formation indicated a high *K*_m_ for SN30000 (974 ± 71 μM) in mouse liver S9, and high sensitivity to O_2_ with ∼50% inhibition at 0.2% O_2_ gas phase, (∼1.87 μM O_2_ in solution at equilibrium). The latter estimate is similar to that for 50% inhibition of SN30000 cytotoxicity in stirred suspensions of HT29 tumor cells [1.14 ± 0.24 μM O_2_ in solution; (Hicks et al., 2010)]. Importantly, the S9 studies demonstrate substantial capacity for one-electron (i.e., oxygen-inhibitable) reduction in liver. Given the lack of urinary excretion of SN30000 in mice (with only ∼5% of injected dose excreted by 24 h), and prominence of reduced metabolites in plasma and tissues, the reductive metabolism route is identified as the major clearance mechanism in mice. However, the locale of this bioreductive metabolism is not clear. The predicted *in vivo* hepatic clearance via reduction to M14 based on intrinsic clearance by anoxic liver S9 (3.75–5.01 L/h/kg) is similar to measured SN30000 clearance in mice at high dose (CL of 5.04 L/h/kg at the dose of 78 mg/kg, Supplementary Table 12). Given that only a small fraction of mouse liver is hypoxic enough to activate the 2-nitroimidazole hypoxia probe pimonidazole (Arteel et al., 1995; Gu et al., 2011a), either the S9 model misses an important component of SN30000 activation (e.g., higher proportion of 2-electron reduction *in vivo* or reduction by mitochondria) or extrahepatic metabolism also plays a major role.

Given this high rate of oxygen-inhibited metabolism, we sought to minimize post-mortem metabolism during evaluation of the biodistribution and PK of SN30000. Post-mortem activation has also been noted as a significant challenge in the case of the 2-nitroimidazole HAP RSU-1069 (Olive et al., 1987). Rapid post-mortem bioreductive metabolism of SN30000 in mouse liver relative to HT29 tumors (Supplementary Figure 6) emphasizes high hepatic reductive capacity. We minimized this artifact by rapidly sampling blood then liver followed by other tissues after cervical dislocation. It is unlikely that the problem has been entirely eliminated for the tissue samples, but is not expected to influence the plasma concentration estimates as demonstrated in Supplementary Figure 7.

The similarity of PK in mice with and without large HT29 tumors (**Figure 2**) demonstrates that despite severe hypoxia in these tumors, they do not make a significant contribution to whole-body bioreduction of SN30000. This again emphasizes the major role of normal tissues in SN30000 metabolism. This initial PK study also demonstrated that concentrations of SN30000 are similar in brain and plasma, with higher relative concentrations of M14 and M13 in brain whilst M18 was not detected (**Figure 2C**). We showed that dosing with M14 itself rather than SN30000 gave even higher brain/plasma M14 concentration ratios (**Figure 3**) demonstrating that M14 partitions from plasma rather than being generated by bioreduction in the brain. These studies establish that SN30000 and M14 cross the blood–brain barrier efficiently, which signals the potential utility of SN30000 for targeting hypoxia in brain tumors.

The dose dependence of SN30000 PK in mice is clearly non-linear (**Figures 7, 8**). A plausible mechanism of this non-linearity is the marked hypothermia induced by M14, and is qualitatively consistent with the observed temperature dependence of one-electron reduction of SN30000 by mouse liver S9 preparations (**Figure 6**). The reduced clearance of SN30000 at high doses is mirrored by the increased plasma half-life of co-administered mannitol, which returns to normal values by 16 h (**Figure 10**) suggesting a reversible physiological change such as hypothermia rather than the irreversible kidney damage noted in the histopathology studies. This provisional 2-compartment PK model also suggests a decrease in first pass metabolism with dose. While a model could have been fitted using traditional concentration-dependent metabolism, the high *K*_m_ for liver S9 relative to plasma SN30000 concentrations (*C*_max_ ∼ 100 μM), together with the temperature effect on mannitol clearance argues against such an interpretation here. This demonstrates the utility of co-administration of tracer compounds to aid interpretation.

In addition to its implications for the toxicokinetics of SN30000, the present study shows extensive normal tissue bioreduction of SN30000 in mice. Other classes of HAPs such as the nitrobenzamide PR-104 (Gu et al., 2011a), the tertiary amine *N*-oxide AQ4N (Loadman et al., 2001) and the 2-nitroimidazole evofosfamide (Jung et al., 2012) also show appreciable metabolic activation in normal tissues. Thus, despite demonstrable selectivity for hypoxic relative to oxic cells in tumors, many normal tissues appear to support significant activation of HAPs from diverse chemical classes. We thus infer that the tumor selectivity of HAPs depends on the relative insensitivity of most normal tissues to the resulting cytotoxins, as well as tumor hypoxia.

## Author Contributions

Conception and design: YG, DE, JW, AH, WW, and KH. Conducted experiments: YG, TC, JJ, JW, HL, CL, WW, KH, MH, and ND. Contributed new reagents: MH. Analysis and interpretation of data: YG, JJ, ND, DE, WW, and KH. Writing, review or revision of the manuscript: YG, JJ, FP, DE, WW, and KH.

## Conflict of Interest Statement

The authors declare that the research was conducted in the absence of any commercial or financial relationships that could be construed as a potential conflict of interest.
