# Supplementary material for: Reductive Metabolism Influences the Toxicity and Pharmacokinetics of the Hypoxia-Targeted Benzotriazine Di-Oxide Anticancer Agent SN30000 in Mice
Source: Front Pharmacol. 2017 Aug 11;8:531. doi: 10.3389/fphar.2017.00531 (PMC5554537; doi:10.3389/fphar.2017.00531)
Supplement: Supplementary file 1 [file Presentation_1.PDF]

Supplementary Material

Bioreductive metabolism and pharmacokinetics of the hypoxia-targeted benzotriazine di-oxide SN30000 in mice

Yongchuan Gu, Tony T-A Chang, Jingli Wang, Jagdish K. Jaiswal, David Edwards, Noel J. Downes, H.D. Sarath Liyanage, Anthony J.R. Hickey, Michael P. Hay, William R. Wilson* and Kevin O. Hicks.

*** Correspondence:** wr.wilson@auckland.ac.nz

TABLE OF CONTENTS Pg

Supplementary Data:

Synthesis of M18 …3

Purification of M18 from mouse plasma and comparison with synthetic standard …4

Validation of LC-MS/MS method for quantitation of SN30000, M14 and M13 in mouse plasma and liver homogenates …4

Supplementary Tables:

Supplementary Table 1. Intra- and inter-day precision and accuracy, recovery and matrix effect for SN30000 and metabolites M14 and M13 in mouse plasma. …6

Supplementary Table 2. Intra- and inter-day precision and accuracy, recovery and matrix effect for SN30000 and metabolites M14 and M13 in mouse liver homogenates. …7

Supplementary Table 3. Stability of SN30000 and metabolites M14 and M13 in mouse plasma. …8

Supplementary Table 4. Stability of SN30000 and metabolites M14 and M13 mouse liver homogenates. …9

Supplementary Table 5. Antiproliferative potency of SN30000, M14 and M18 against HCT116 cells. …10

Supplementary Table 6. Mouse toxicity SN30000 and metabolites M14 and M18: survival and body weight loss …11

Supplementary Table 7. Mouse toxicity of SN30000 and M14: Histopathology findings …12

Supplementary Table 8. Non-compartmental PK parameters for SN30000, and its major metabolites following i.p. administration of SN30000 at its MTD (186 mg/kg) to HT-29 tumour-bearing and non-tumour-bearing NIH-III mice …13

Supplementary Table 9**.** Non-compartmental PK parameters of, M13, M14 and M18 in plasma, liver, and brain in female NIH-III nude mice treated with M14 at 135 mg/kg by i.p administration. …14

Supplementary Table 10. Inhibition of mouse brain acetylcholinesterase activity by SN30000 and related compounds …15

Supplementary Table 11. Protein binding of SN30000, M14 and M18 in mouse plasma …16

Supplementary Table 12. Non-compartmental PK parameters for SN30000 and its major metabolites following i.p. bolus administration of SN30000 to female NIH-III mice …17

Supplementary Table 13. Compartmental model parameters for SN30000, its metabolites and mannitol. …18

Supplementary Table 14. Non-compartmental parameters for ^{3}H-mannitol plasma pharmacokinetics after SN30000 dosing …19

Supplementary Figures:

Supplementary Figure 1. Representative chromatogram of SN30000 metabolites in NIH-III mice …20

Supplementary Figure 2. Absorbance spectra and mass spectra of SN30000 metabolites …21

Supplementary Figure 3, Initial rates of core temperature decrease in NIH-III mice post-mortem: comparison with initial temperature change following treatment with benzotriazine oxides at their MTD. …28

Supplementary Figure 4. Time-dependent changes in oxygen consumption and H_2O_2 formation rates induced by SN30000 in rotenone-inhibited CHO/POR cell suspensions, monitored with an OXYBOROS O2K oxygraph …29

Supplementary Figure 5. Effects of SN30000, M14 and TPZ on oxygen consumption rates (OCR) of human cervical carcinoma cell lines, determined using a Seahorse XFe96 Analyser …31

Supplementary Figure 6. Post-mortem metabolism of SN30000 and M14: time dependence of tissue concentrations …33

Supplementary Figure 7. Post-mortem metabolism of SN30000 in female NIH-III mice: dependence on sequence of tissue sampling …34

Supplementary Figure 8. Concentration-time profiles for SN30000 administered i.v. to female NIH-III mice at a range of doses. …35

Supplementary Figure 9. Plasma and tissue concentration-time profiles for M14, fitted using a compartmental model …36

Supplementary references …37

SUPPLEMENTARY DATA:

Syntheses of 3-(2-carboxyethyl)-7,8-dihydro-6H-indeno[5,6-e][1,2,4]triazine 1-oxide (M18)

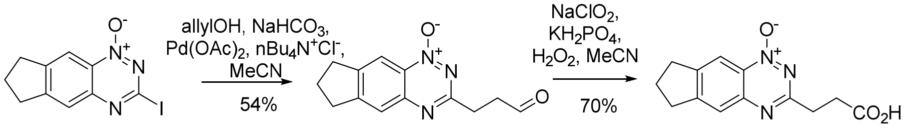

S1 S2 M18

An authentic sample of M18 was prepared by palladium-mediated Sonogashira coupling of the iodide S**1** (Hay et al., 2008) to give aldehyde S**2**. This was oxidised with sodium perchlorite in aqueous hydrogen peroxide to give M18. Analyses were carried out in the Campbell Microanalytical Laboratory, University of Otago, Dunedin, NZ. The products were analysed by reverse-phase HPLC, (Altima C18 5 µm column, 150 × 3.2 mm; Alltech Associated, Inc., Deerfield, IL) using an Agilent HP1100 equipped with a diode-array detector. Mobile phases were gradients of 80% acetonitrile/20% H_2O (v/v) in 45 mM ammonium formate at pH 3.5 and 0.5 mL/min. Final compound purity was determined by monitoring at 330 ± 50 nm and was >95%. Melting points were determined on an Electrothermal 2300 Melting Point Apparatus. NMR spectra were obtained on a Bruker Avance 400 spectrometer at 400 MHz for ^{1}H and 100 MHz for ^{13}C spectra. Spectra were obtained in $CDCl_3$ and were referenced to Me_4Si. Chemical shifts and coupling constants were recorded in units of ppm and Hz, respectively. Assignments were determined using COSY, HSQC, and HMBC two-dimensional experiments. Low resolution mass spectra were gathered by direct injection of methanolic solutions into a Surveyor MSQ mass spectrometer using an atmospheric pressure chemical ionization (APCI) mode with a corona voltage of 50 V and a source temperature of 400 °C. High resolution mass spectra (HRMS) were measured on a Bruker microTOF-QII Hybrid Quadrupole Time of Flight (TOF-Q) mass spectrometer interfaced with either an Electrospray Ionization (ESI) or Atmospheric Pressure Chemical Ionization (APCI) probe allowing positive or negative ions detection. Solutions in organic solvents were dried with anhydrous $MgSO_4$. Solvents were evaporated under reduced pressure on a rotary evaporator. Thin-layer chromatography was carried out on aluminium-backed silica gel plates (Merck 60 F_{254}) with visualization of components by UV light (254 nm) or exposure to I_2. Column chromatography was carried out on silica gel (Merck 230–400 mesh).

3-(1-Oxido-7,8-dihydro-6*H*-indeno[5,6-*e*][1,2,4]triazin-3-yl)propanal (S2). $Pd(OAc)_2$ (516 mg, 2.28 mmol) was added to a degassed mixture of iodide S**1** (7.15 g, 22.8 mmol), $NaHCO_3$ (4.23 g, 50.0 mmol), tetrabutylammonium chloride (6.35 g, 22.8 mmol) and allyl alcohol (7.77 mL, 114 mmol) in acetonitrile (250 mL) and the mixture was stirred at reflux

temperature for 1 h. The mixture was cooled, silica gel was added and the slurry stirred at 20 °C for 5 min. The mixture was evaporated to dryness and the powder was applied to a short pad of silica gel, eluting with a gradient (0–30%) of EtOAc/pet. ether, to give a light brown solid. This material was further purified by chromatography, eluting with 30% EtOAc/pet. ether, to give aldehyde S**2** (3.54 g, 64%) as an off white solid: mp (EtOAc/pet. ether) 72–74 °C; ^{1}H NMR δ 9.93 (t, *J* = 0.9 Hz, 1 H, CHO), 8.25 (s, 1 H, H-9), 7.73 (s, 1 H, H-5), 3.35 (t, *J* = 7.0 Hz, 2 H, CH_2), 3.07–3.14 (m, 6 H, H-6, H-8, CH_2), 2.21 (p, *J* = 7.5 Hz, 2 H, H-7); ^{13}C NMR δ 200.4, 163.9, 154.8, 149.1, 147.2, 132.3, 122.7, 114.2, 40.5, 33.1, 32.8, 29.4, 25.7; MS *m/z* 244.2 (MH^+, 100%); HRMS (CI, CH_3OH) calcd for $C_{13}H_{14}N_3O_2$ (MH^+) *m/z* 244.1086, found 244.1088. Anal. calcd for $C_{13}H_{13}N_3O_2$: C, 64.2; H, 5.4; N, 17.3. Found: C, 63.9; H, 5.5; N, 17.0%.

3-(2-Carboxyethyl)-7,8-dihydro-6*H*-indeno[5,6-e][1,2,4]triazine 1-Oxide (M18). A solution of $NaClO_2$ (222 mg, 1.96 mmol) in water (3 mL) was added drop-wise to a stirred mixture of S**2** (341 mg, 1.40 mmol) in MeCN (50 mL) and NaH_2PO_4 (48 mg, 0.35 mmol) in water (10 mL) and 35% H_2O_2 (0.14 mL, 1.40 mmol) at 5 °C and the mixture was stirred at 5 °C for 4 h. The mixture was acidified with aqueous HCl (1 M) and extracted with EtOAc (3 × 50 mL). The combined organic fraction was dried and the solvent evaporated. The residue was crystallised from EtOAc/pet. ether to give the acid (261 mg, 72%) as a white powder: mp 215 °C (dec.); ^{1}H NMR δ 8.24 (s, 1 H, H-9), 7.74 (s, 1 H, H-5), 3.34 (br t, *J* = 7.0 Hz, 2 H, CH_2), 3.08–3.14 (m, 4 H, H-6, H-8), 3.01 (t, *J* = 7.0 Hz, 2 H, CH_2), 2.22 (p, *J* = 7.4 Hz, 2 H, H-7), OH not observed; ^{13}C NMR δ 176.4, 164.0, 155.2, 149.4, 147.2, 132.6, 122.8, 114.6, 33.3, 33.0, 31.4, 31.0, 25.9; MS *m/z* 260.3 (MH^+, 100%); Anal. calcd for $C_{13}H_{13}N_3O_3$: C, 60.22; H, 5.05; N, 16.21. Found: C, 60.46; H, 5.08; N, 16.34%.

Purification of M18 from mouse plasma and comparison with synthetic standard

Plasma was harvested from C57Bl/6 mice 1 hr after i.p. dosing with M14 (135 mg/kg), extracted with 3 vol ice-cold MeOH and centrifuged (13,000 *g*, 4°C) for 10 min. The resulting supernatant was evaporated (35°C, 1 hr) using a refrigerated CentriVap Vacuum Concentrator (Labconco, Morriston, NJ, USA). Solids were dissolved with 1% MeOH/0.02% acetic acid, loaded onto an Agilent Bond Elut C8 1 mL cartridge (Lake Forrest, CA, USA) which was rinsed with 2% MeOH/0.02% acetic acid before eluting with 0.02% acetic acid in MeOH. The eluent was evaporated to dryness as above and reconstituted in 100% MeOH. M18 was isolated from this extract by fraction collection from an Agilent 1100 HPLC using an Altima C18 column (150 × 4.6 mm, 5 µm particle size) at 25°C, with a gradient of MeCN in 0.02% acetic acid/water at 1 ml/min and a run time of 23 min. The eluate was monitored at 252 nm and the M18 fraction was collected between 15.5-16.0 min and evaporated to dryness for NMR analysis. The isolated product was shown to be identical with the M18 synthetic standard with respect to HPLC retention time, UV and mass spectra, and NMR spectroscopy.

Validation of LC-MS/MS method for quantitation of SN30000, M14 and M13 in mouse plasma and liver homogenates

Chromatographic peaks of analytes and internal standards were identified on the basis of their retention times and MRM responses. Linearity of calibration curves based on peak area ratios (target/internal standard) were assessed by weighted (1/x) least squares regression analysis

using octa-deuterated internal standards of SN30000, M13 and M14 synthesised as previously reported (Wang et al., 2012). Intra- and inter-day precision (expressed as relative standard deviation (RSD) and accuracy (expressed as percentage of the nominal value) were determined by analysis of replicates (n=6) of low and high quality control (QC) samples on three different days. The lower limit of quantitation (LLOQ) was defined as the lowest concentration providing RSD and accuracy < 20%. Recovery from plasma and liver homogenates was tested at the three QC levels. The recoveries were evaluated by comparing extracted samples with blank extracted solutions spiked with analytes of the same amount. Matrix effects, defined as the change in response due to the presence of other substances in the samples, were evaluated by comparing the analytes diluted into the blank plasma or liver extracted solution with that of the same stock solutions diluted in water. Three different concentration levels (low, medium and high QC) were evaluated by analysing three samples at each level. Accuracy, precision, recovery and matrix effects were all acceptable over the concentration ranges evaluated for plasma (Supplementary Table 1) and for liver homogenates (Supplementary Table 2).

Stability of the analytes in mouse plasma (Supplementary Table 3) or liver homogenate (Supplementary Table 4) was confirmed during holding of QC samples on ice for up to 2 h under normal laboratory lighting, although there was a trend towards loss of SN30000 in plasma samples held in clear microcentrifuge tubes which may reflect the known photosensitivity of the compound (Gu et al., 2014). This loss was reduced by use of red microcentrifuge tubes, and eliminated by black tubes. Stability in both matrices was also confirmed for one month storage at -80°C and for three freeze-thaw cycles, while MeOH-deproteinised samples were stable for 12 h at 4°C in the autosampler (Supplementary Tables 3 and 4).

SUPPLEMENTARY TABLES

SUPPLEMENTARY TABLE 1. Intra- and inter-day precision and accuracy, recovery and matrix effect for SN30000 and metabolites M14 and M13 in mouse plasma.

	Nominal Conc (μM)	Intra-day			Inter-day			Recovery (%)	No Matrix conc (μM)	Matrix effect (%)
		Measured conc (μM)	Accuracy	RSD(%)	Measured conc (μM)	Accuracy	RSD(%)			
SN30000	50	47.3	94.6	1.51	51.4	102.8	6.50	93.2	52.0	102.7
	5	4.60	92.1	3.16	4.82	96.4	3.84	99.1	4.96	96.7
	0.5	0.45	89.2	1.88	0.47	93.6	4.59	94.9	0.52	91.6
	0.01[a]	0.012	118.0	2.23	0.011	114.1	14.2	-	-	-
M14	25	24.8	99.2	1.23	25.9	103.5	3.98	98.9	24.1	104.1
	2.5	2.32	92.6	3.12	2.38	95.3	2.93	99.2	2.32	98.0
	0.25	0.22	87.2	1.32	0.23	90.7	3.89	98.5	0.24	91.4
	0.005[a]	0.0058	115.5	8.28	0.0059	117.9	13.9	-	-	-
M13	10	9.81	98.1	1.66	10.45	104.5	5.49	100.9	9.56	85.4
	1	0.919	91.9	2.53	0.96	96.5	4.25	101.7	0.92	92.0
	0.1	0.089	89.4	1.99	0.09	90.4	5.95	101.8	0.092	103.1
	0.002[a]	0.0024	118.8	13.38	0.0023	117.2	16.8	-	-	-

[a]Lower limit of quantitation

SUPPLEMENTARY TABLE 2. Intra- and inter-day precision and accuracy for SN30000 and its metabolites in mouse liver homogenates

	Nominal Conc (µM)	Intra-day			Inter-day			Recovery (%)	No matrix conc (µM)	Matrix effect (%)
		Measured conc (µM)	Accuracy	RSD(%)	Measured conc (µM)	Accuracy	RSD(%)			
SN30000	50	47.6	95.3	5.75	47.9	95.8	8.93	93.9	48.1	104.4
	5	4.54	90.8	5.27	4.74	94.7	5.29	97.7	4.64	112.2
	0.5	0.49	97.6	3.64	0.499	99.8	4.27	100.9	0.49	103.8
	0.01[a]	0.0081	81.1	4.95	0.0097	97.4	16.88	-	-	-
M14	25	25.2	100.8	4.89	24.6	98.3	10.20	99.0	25.2	100.8
	2.5	2.52	100.9	5.38	2.50	100.0	6.26	100.7	2.39	106.1
	0.25	0.24	96.6	3.73	0.25	98.3	3.80	103.5	0.24	96.5
	0.005[a]	0.0058	115.1	9.84	0.0059	117.8	14.24	-	-	-
M13	10	9.74	97.4	5.19	9.92	99.2	8.65	95.1	9.88	104.4
	1	1.00	100.1	5.15	1.04	104.0	5.63	100.8	0.94	112.2
	0.1	0.094	94.1	4.83	0.100	100.3	6.48	102.1	0.095	103.8
	0.002[a]	0.0018	91.2	18.18	0.0022	108.2	18.62	-	-	-

[a]Lower limit of quantitation

SUPPLEMENTARY TABLE 3: Stability of SN30000 and metabolites M14 and M13 in mouse plasma

	Vial[a]	Nominal conc (µM)	SN30000			M14			M13		
			0.5	5	50	0.25	2.5	25	0.1	1	10
1 h on ice	Clear	Measured conc (µM)	0.46	4.60	46.39	0.23	2.45	24.37	0.09	0.96	9.64
		RSD (%)	2.89	2.41	5.02	3.69	1.56	1.50	3.36	2.40	1.91
		Bias (%)	-7.83	-7.91	-7.21	-6.07	-2.06	-2.50	-7.05	-3.76	-3.61
	Red	Measured conc (µM)	0.48	4.64	47.57	0.24	2.39	25.07	0.09	0.95	10.01
		RSD (%)	2.20	1.09	3.27	2.61	0.87	1.73	2.04	0.95	0.49
		Bias (%)	-3.33	-7.29	-4.85	-3.25	-4.29	0.26	-5.72	-5.29	0.10
	Black	Measured conc (µM)	0.50	4.64	52.5	0.23	2.25	25.80	0.09	0.93	10.59
		RSD (%)	0.85	1.36	5.56	1.07	2.26	2.86	0.87	2.47	2.76
		Bias (%)	-0.80	-7.17	5.03	-6.07	-9.84	3.20	-7.00	-7.17	5.90
2 h on ice	Clear	Measured conc (µM)	0.43	4.38	42.53	0.24	2.51	24.39	0.09	0.95	9.10
		RSD (%)	2.03	1.05	4.50	1.85	1.28	1.17	2.67	1.37	1.01
		Bias (%)	-14.4	-12.3	-14.9	-5.90	0.51	-2.43	-9.85	-4.92	-8.95
	Red	Measured conc (µM)	0.45	4.37	45.94	0.24	2.48	25.17	0.09	0.95	9.61
		RSD (%)	2.83	2.17	1.89	2.06	2.90	2.32	2.91	3.13	1.83
		Bias (%)	-10.09	-12.57	-8.13	-3.27	-0.72	0.70	-7.29	-4.71	-3.91
	Black	Measured conc (µM)	0.51	5.01	51.16	0.24	2.43	24.42	0.10	1.01	10.06
		RSD (%)	3.33	2.69	2.32	2.31	2.66	3.09	3.62	3.09	3.47
		Bias (%)	1.31	0.11	2.32	-2.23	-2.62	-2.33	-0.52	1.13	0.55
Three freeze-thaw cycles		Measured conc (µM)	0.50	4.86	53.36	0.24	2.42	25.98	0.10	0.97	10.44
		RSD (%)	2.55	3.41	1.48	1.26	2.29	0.79	1.28	3.21	1.57
		Bias (%)	0.56	-2.87	6.72	-4.07	-3.19	3.92	-4.05	-2.61	4.39
1 month at -80°C		Measured conc (µM)	0.48	4.92	53.90	0.23	2.42	26.65	0.10	1.00	11.05
		RSD (%)	3.78	1.52	7.80	6.84	3.19	6.60	3.62	0.13	10.50
		Bias (%)	1.32	1.44	2.04	1.83	1.63	1.59	1.68	1.94	1.85
12 h autosampler at 4°C[b]		Measured conc (µM)	0.50	4.99	53.97	0.24	2.48	26.29	0.10	0.99	10.54
		RSD (%)	6.06	2.58	4.27	5.87	3.53	3.47	6.44	3.59	4.24
		Bias (%)	-0.12	-0.22	7.93	-3.45	-1.00	5.16	-3.42	-1.26	5.41

[a] 1.5 ml Eppendorf microcentrifuge tubes.

[b] Plasma samples deproteinised in 3 vol MeOH

SUPPLEMENTARY TABLE 4. Stability of SN30000 and metabolites M14 and M13 mouse liver homogenates.

Condition	Nominal conc (µM)	SN30000			M14			M13		
		0.5	5	50	0.25	2.5	25	0.1	1	10
1h on ice [a]	Measured conc (µM)	0.51	5.24	53.43	0.26	2.54	25.76	0.11	1.10	10.78
	RSD (%)	1.95	3.01	1.42	2.01	5.02	3.26	2.65	4.61	3.75
	Bias (%)	2.46	4.72	6.87	5.21	1.78	3.05	11.64	10.33	7.77
2h on ice	Measured conc (µM)	0.48	5.14	50.60	0.27	2.73	25.19	0.10	1.03	10.11
	RSD (%)	1.81	1.27	4.02	1.72	1.69	1.08	1.55	0.94	1.48
	Bias (%)	-3.26	2.72	1.20	8.20	9.32	0.75	2.48	3.25	1.11
Three freeze-thaw cycles	Measured conc (µM)	0.45	4.59	52.39	0.24	2.36	26.35	0.093	0.96	10.56
	RSD (%)	6.98	5.32	2.38	6.59	4.31	2.41	2.84	4.41	1.94
	Bias (%)	-10.74	-8.29	4.78	-4.84	-5.62	5.39	-7.15	-4.26	5.64
1 month at -80°C	Measured conc (µM)	0.52	4.77	47.06	0.25	2.35	23.54	0.11	1.03	9.12
	RSD (%)	3.57	4.53	5.88	1.01	5.81	5.83	6.86	2.59	8.84
	Bias (%)	1.98	4.40	4.71	1.76	4.04	5.13	1.60	4.04	5.05
12 h autosampler at 4°C [b]	Measured conc (µM)	0.52	5.14	53.37	0.26	2.64	26.30	0.11	1.11	10.64
	RSD (%)	2.82	3.42	2.51	3.00	2.67	2.00	2.96	3.47	1.31
	Bias (%)	3.36	2.86	6.75	3.86	5.73	5.22	7.07	10.75	6.40

[a] Samples in clear 1.5 ml Eppendorf microcentrifuge tubes

[b] Liver homogenates deproteinised in 3 vol MeOH

SUPPLEMENTARY TABLE 5. Antiproliferative potency of SN30000 and its major metabolites following 4 h exposure of HCT116 cells under aerobic or anoxic conditions.

Gas phase	IC_{50} (µM) [a]		
	SN30000	1-oxide (M14)	1-oxide acid (M18)
Aerobic	148 ± 20 (5) [b]	600 ± 60 (4)	>1670 (2)
Anoxic	1.7 ± 0.4 (5)	900 ± 100 (4)	>1670 (2)

[a] HCT116 cells, originally from ATCC, were authenticated by short tandem repeat profiling and confirmed mycoplasma-free by PCR/ELISA assay. Cells were passaged in Alpha MEM with 5% foetal bovine serum without antibiotics. *In vitro* cytotoxicity of SN30000 and metabolites was measured as the inhibition of cell proliferation as described previously (Gu et al., 2009). Briefly, cells were exposed to compounds for 4 h under aerobic (20% O_2) or anoxic (Bactron anaerobic chamber) conditions and then grown in an aerobic 5% CO_2 incubator for 5 d before staining with sulforhodamine B. IC_{50} values were determined by interpolation (nonlinear regression to the standard Hill equation) as the drug concentration reducing staining to 50% of controls on the same plate.

[b] Values are mean ± SEM for the number of independent experiments shown in parentheses.

SUPPLEMENTARY TABLE 6. Mouse toxicity SN30000 and metabolites M14 and M18: survival and body weight loss following single i.p. doses to NIH-III mice.

Cmpd	Dose (μmol/kg)	Male			Female		
		Survival[a]	Body weight nadir		Survival[a]	Body weight nadir	
			Day	Weight loss (%)[b]		Day	Weight loss (%)[b]
SN30000[c]	562	7/7	1	4.2 ± 1.2	6/6	1	8.4 ± 1.7
	750	2/3	1	12.1 ± 3.8	1/2	2	14.4
	1000	1/2	1	13.3	0/1	-[d]	-
	1333	1/2	1	11.6			
M14	562	3/3	1	0.7 ± 0.5	3/3	3	2.6 ± 0.4
	750	0/2	ND	ND	0/1	ND	ND
	1000	1/1	1	2.0	ND		
M18	562	ND			3/3	- [e]	-

[a] Survival reported as a fraction of total number of animal treated at a given dose, with an observation time of 14 days. Deaths were predominantly due to humane termination because of persistent clinical signs or body weight loss > 15% of pretreatment values.

[b] Body weight loss at nadir, relative to pre-treatment values. Values are mean and errors are SEM.

[c] Data from Gu et al., 2014

[d] Mouse died <24 hr after dosing.

[e] No decrease in body weight

SUPPLEMTENTAL TABLE 7. Mouse toxicity of SN30000 and M14: Histopathology findings

Male and female NIH-III mice aged 6-10 weeks were given a single i.p. dose of SN30000 (186, 248 or 331 mg/kg), or metabolite M14 (177 mg/kg, equimolar to SN30000 at 186 mg/kg). After 15-17 days, selected tissues/organs were collected from surviving animals at necropsy and from a single untreated control for each sex, fixed in 10% neutral buffered formalin at room temperature for 24-48 hr, transferred to 70% ethanol and subsequently paraffin embedded, sectioned and H&E stained for light microscopy. The following tissues/organs were examined: skin, testes, eyeball, brain, liver, lung, stomach, duodenum, heart, spleen, pancreas, colon, femur and joint (incl marrow), skeletal muscle (psoas) and kidney. Results are reported only for positive findings. There were no positive findings for M14.

Cmpd	Tissue	Dose (mg/kg)	Sex	Number examined	Findings
SN30000	Kidney	186	M	3	Basophilic cortical tubules (1)[a]; tubular epithelial necrosis (1), cortical tubular vacuolation (1).
			F	3	Basophilic cortical tubules (2); karyomegaly in tubular epithelium (3); tubular epithelial necrosis (3).
		248	M	2	Basophilic cortical tubules (1)[a]; karyomegaly in tubular epithelium (1); tubular epithelial necrosis (1).
			F	2	Basophilic cortical tubules (2); karyomegaly in tubular epithelium (2); tubular epithelial necrosis (2).
		331	M	1	Basophilic cortical tubules (1); karyomegaly in tubular epithelium (1); tubular epithelial necrosis (1).
	Lungs	186	F	3	Hyperplasia/dysplasia in terminal bronchioles (1)
		248	M	2	Hyperplasia/dysplasia in terminal bronchioles (2)
			F	2	Hyperplasia/dysplasia in terminal bronchioles (2)
	Testes	248	M	1	Degeneration of germinal epithelium (1)

[a] Number of animals with abnormality.

SUPPLEMENTARY TABLE 8. Non-compartmental PK parameters for SN30000, and its major metabolites following i.p. administration of SN30000 at its MTD (186 mg/kg) to HT29 tumour-bearing and non-tumour-bearing NIH-III mice. Concentration-time curves are shown in Fig. 2.

Compound	Parameter	Plasma	Liver	Brain	Tumour
SN30000	AUC[b] (μmol.hr/kg)	156<br>(159)[a]	147<br>(164)	158<br>-	-<br>(111)
	Terminal $T_{1/2}$ (min)	32.7<br>(33.9)	44.3<br>(35.7)	26.4<br>-	-<br>(36.2)
	C_{max} (μmol/kg)	210 ± 9<br>(217± 18)	164 ± 38<br>(172 ± 8)	217 ± 12<br>-	-<br>(69.9 ± 12.1)
M14	AUC[b] (μmol.hr/kg)	26.4<br>(27.6)	350<br>(305)	126<br>-	-<br>(136)
	Terminal $T_{1/2}$ (min)	37.8<br>(40.6)	40.5<br>(42.7)	32.1<br>-	-<br>(94.8)
	C_{max} (μmol/kg)	36.1 ± 5.5<br>(33.1 ± 2.6)	469 ± 27<br>(371 ±26)	162 ± 0<br>-	-<br>(46.0 ± 3.9)
M13	AUC[b] (μmol.hr/kg)	13.0<br>(14.9)	158<br>(158)	46.7<br>-	-<br>(57.7)
	Terminal $T_{1/2}$ (min)	42.4<br>(53.2)	42.1<br>(52.4)	34.9<br>-	-<br>(126)
	C_{max} (μmol/kg)	14.5 ± 2.6<br>(13.3 ± 1.0)	178.7 ± 8.1<br>(155.4 ±8.4)	52.8 ± 4.1<br>-	-<br>(15.5 ± 2.2)
M18	AUC[c] (μmol.hr/kg)	349<br>(322)	107<br>(92)	-	34.8
	Terminal $T_{1/2}$ (min)	173<br>(960)	183<br>(392)	-	-
	C_{max} (μmol/kg)	119 ± 3<br>(139 ± 6)	53.5 ± 4.0<br>(46.1 ±5.2)	-	(17.6 ± 1.8)

[a] PK parameters for tumour bearing mice are in parentheses. Brain PK not determined in tumour-bearing mice.
[b] AUC estimated from extrapolation to infinity. Values are from terminal bleed sampling of plasma followed by liver and brain (3 mice per time point).
[c]AUC 0-3hr

SUPPLEMENTARY TABLE 9**.** Non-compartmental PK parameters of M14, M13 and M18 in plasma, liver, and brain in female NIH-III nude mice treated with M14 at 135 mg/kg by i.p administration. Values are from terminal sampling of plasma followed by liver then brain from each individual. C_{max} values are mean and SEM for 4 animals.

	PK parameters	Plasma	Liver	Brain
M14	AUC (µmol.hr/kg)	36.6	131.1	108.3
	Terminal T1/2 (min)	30.8	25.1	20.1
	Cmax (µM)	95.6 ± 9.8	319.4 ± 46.1	260.2 ± 29.0
M13	AUC (µmol.hr/kg)	1.79	14.9	5.3
	Terminal T1/2 (min)	40	32.7	26.0
	Cmax (µmol/kg)	4.0 ± 0.63	34.1 ± 5.4	11.0 ± 1.6
M18	AUC (µmol.hr/kg)	349	93.3	-
	Terminal T1/2 (min)	182	144.3	-
	C_{max} (µmol/kg)	144 ± 5.3	40.0 ± 3.0	-

SUPPLEMENTARY TABLE 10: Inhibition of mouse brain acetylcholinesterase activity[a] by SN30000 and related compounds

Compound	IC_{50} (μM)	SE
SN30000	131	7
M14	58.3	3.1
M18	>> 300	-
TPZ	>> 300	-
TPZ-1-oxide	>> 300	-

[a]The acetylcholinesterase inhibition assay is based on Obregon et al., 2005. Mouse brain homogenate was prepared in potassium phosphate buffer (100 mM, pH 7.4), which was pre-incubated with dithiobisnitrobenzoate (final concentration 1 mM) at 37°C for 10 min. After adding acetylthiocholine (final concentration 0.6 mM), the formation of 5-thio-2-nitrobenzoate was measured by monitoring absorbance (Spectramax- M2 spectrophotometer) at 412 nm every 30 s for 10 min. Negative controls (DMSO or saline) and positive control (paraoxon 10 μM, which provided 96 ± 2% inhibition) was included with each determination. IC_{50} values are based on 3-4 determinations per compound.

SUPPLEMENTARY TABLE 11: Protein binding of SN30000, M14 and M18 at the indicated concentrations in NIH-III mouse plasma, measured by equilibrium dialysis[a].

Concentration (μM)	Percent Protein Bound (Mean ± SD)[b]		
	**SN30000**	**M14**	**M18**
10	21.93 ± 0.17	56.63 ±0 .06	91.77 ± 0.01
100	26.68 ± 0.04	65.81 ± 0.02	90.32 ± 0.01

[a] Protein binding was evaluated by equilibrium dialysis using a high throughput plate with 12-14KDa dialysis membrane (HTDialysis, Gales Ferry, CT) as described by Banker et al., 2003, with modification. Briefly, 100 µL samples of plasma containing SN30000 or metabolites (M14 and M18) were dialysed against 100 µL PBS (pH 7.4) in an incubator (Innova-42, New Brunswick Scientific, US) at 37°C with shaking at 80 rpm for 6 h. To achieve matrix match, 10 µL of blank plasma was added to 90 µL of the PBS sample and 90 µL of PBS was added to 10 µL of the plasma sample. Proteins were precipitated with 3 vol of ice-cold MeCN, the centrifuged supernatants were mixed with 2 vol 45 mM ammonium formate buffer pH 4.5, and 50 µL samples were analysed by HPLC with absorbance detection.

[b]Values are mean and SD for triplicate samples.

SUPPLEMENTARY TABLE 12. Non-compartmental plasma PK parameters for SN30000 and its major metabolites following i.p. bolus administration of SN30000 to female NIH-III mice (3 mice/group).

		Dose (mg/kg)				
Compound	Parameter	1.86	18.6	78.3	92.8	186
SN30000	AUC (µmol.hr/L)	0.17	2.10	31.5	49.2	155
	T1/2 (min)	20.8	25.0	16.1	19.7	42.5
	Cmax (µmol/L)	0.59 ± 0.10	6.80 ± 0.30	60.8 ± 1.3	77.9 ± 2.1	229 ± 42
	Apparent Cl(L/hr/kg)	33.2	26.8	7.54	5.70	3.55
M14	AUC (µmol.hr/L)	0.02	0.38	7.52	10.8	24.6
	T1/2 (min)	29.5	32.5	20.1	22.9	58.1
	Cmax (µmol/L)	0.053 ± 0.008	1.11 ± 0.08	13.3 ± 3.6	16.8 ± 2.0	23.0 ± 6.6
M13	AUC (µmol.hr/L)	0.0029	0.079	2.64	5.43	14.3
	T1/2 (min)	19.8	39.1	31.8	26.2	53.3
	Cmax (µmol/L)	0.008 ± 0011	0.202 ± 0.023	3.85 ± 1.19	6.63 ± 1.00	9.77 ± 3.07
M18	AUC (µmol.hr/L)	1.06	10.8	51.5	116	204
	T1/2 (min)	102	220	35.5	145	1212
	Cmax (µmol/L)	0.65 ± 0.04	5.44 ± 0.41	23.4 ± 1.2	52.8 ± 1.4	79.5 ± 4.5

SUPPLEMENTARY TABLE 13. Compartmental model parameters for SN30000, its metabolites and mannitol. The model is illustrated schematically in Figure 9.

Parameter type	Parameter	Value	SE%	Units
Absorption rate constants	Ka SN30000	60	Fixed	L/hr
	Kloss for i.p. SN30000	67.48	19.00	L/hr
	Ka 1-Oxide	4.43	18.26	L/hr
Clearances	CL SN30000 to 1-oxide	11.32	7.38	L/hr/kg
	CL 1-Oxide	61.14	15.99	L/hr/kg
	CL 1-Oxide to nor-oxide	15.03	21.40	L/hr/kg
	CL 1-Oxide to M18	3.89	97.58	L/hr/kg
	CL Nor-oxide	76.65	24.77	L/hr/kg
	CL M18	0.23	96.60	L/hr/kg
	CL Mannitol	1.17	3.81	L/hr/kg
Intercompartmental clearances	Clic SN30000	1.12	33.47	L/hr/kg
	Clic 1-Oxide	4.64	41.75	L/hr/kg
	Clic Nor-oxide	9.84	53.48	L/hr/kg
Central compartment volumes	V1 SN30000	1.95	10.9	L/kg
	V1 1-Oxide	2.2	Fixed	L/kg
	V1 Nor-oxide	2.2	Fixed	L/kg
	V1 M18	0.43	97.64	L/kg
	V1 Mannitol	0.39	4.82	L/kg
Peripheral compartment volumes	V2 SN30000	0.64	21.10	L/kg
	V2 1-Oxide	3.51	26.0	L/kg
	V2 Nor-oxide	9.38	38.33	L/kg
Steady state volumes	VSS SN30000	2.60	23.75	L/kg
	VSS 1-Oxide	5.71	26.00	L/kg
	VSS Nor-oxide	11.58	38.33	L/kg
	VSS M18	0.43	97.64	L/kg
	VSS Mannitol	0.39	4.82	L/kg
Temperature effects (N)	CL SN30000 to 1-oxide	0.64	4.81	36%[a]
	Kloss for i.p. SN30000	2.80	41.98	86%
	CL 1-Oxide	2.48	34.98	82%
	CL Nor-oxide	0.38	22.89	23%
	CL Mannitol	0.40	5.03	24%
	V1 1-Oxide	0.48	18.25	28%

[a] These numbers indicate the approximate % suppression of the corresponding parameters per °C.

SUPPLEMENTARY TABLE 14. Non-compartmental parameters of ^{3}H-mannitol for the data shown in Figure 9 where female NIH-III nude mice were dosed with ^{3}H-mannitol at 25µmol/kg by administration either together with the indicated doses of SN30000 i.p. (shown in Table 2) or 16 h after SN30000 dosing i.p. All ^{3}H-mannitol PK parameters are calculated from mannitol concentrations as described in Methods for SN30000 and its metabolites.

	**Mannitol**						
	**Co-administered with SN30000**				**Administered 16hr after SN30000**		
**SN30000 Dose (mg/kg)**	**1.86**	**18.6**	**92.8**	**186**	**0**	**18.6**	**186**
% of MTD	1%	10%	50%	100%	0%	10%	100%
AUC (µM.hr/L)	16.1	17.9	36.2	44.5	17.5	19.6	20.4
T1/2 (min)	14.1	13.1	24.6[a]	26.9[a]	19.7	21.0[b]	23.2[b]
Cmax (µM)	41.2 ±2.87	43.5 ±4.30	52.3 ±2.95	51.0 ±6.72	31.7 ±2.86	36.9 ±4.30	33.2 ±6.79

[a] Significantly different from mannitol T1/2 obtained when mice dosed at 1.86 mg/kg ($p<0.001$).
[b] Not significantly different from untreated group (p=*** and ***, respectively).

SUPPLEMENTARY FIGURES

Supplementary Figure 1. Representative chromatograms for plasma, liver and tumour samples from the same NIH-III mouse 30 min after i.p. dosing with SN30000 at 186 mg/kg (MTD). Absorbance of the HPLC eluate was monitored at 252 nm. Peaks not present in untreated plasma are numbered in order of retention time.

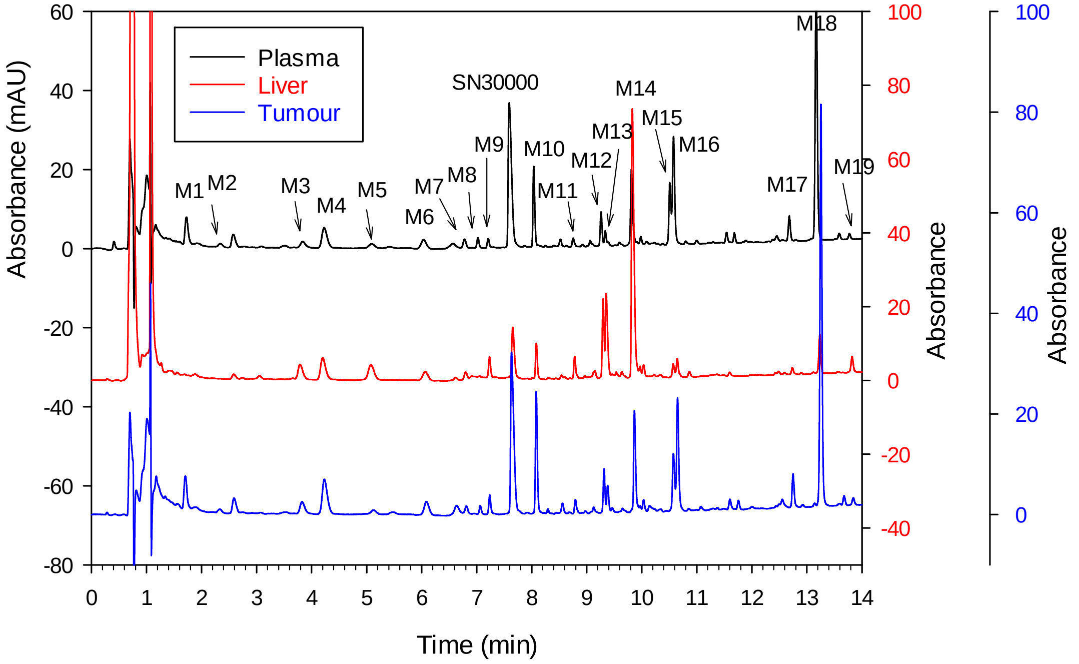

Supplementary Figure 2. Absorbance spectra and mass spectra of SN30000 metabolites

SN30000 M14 M13

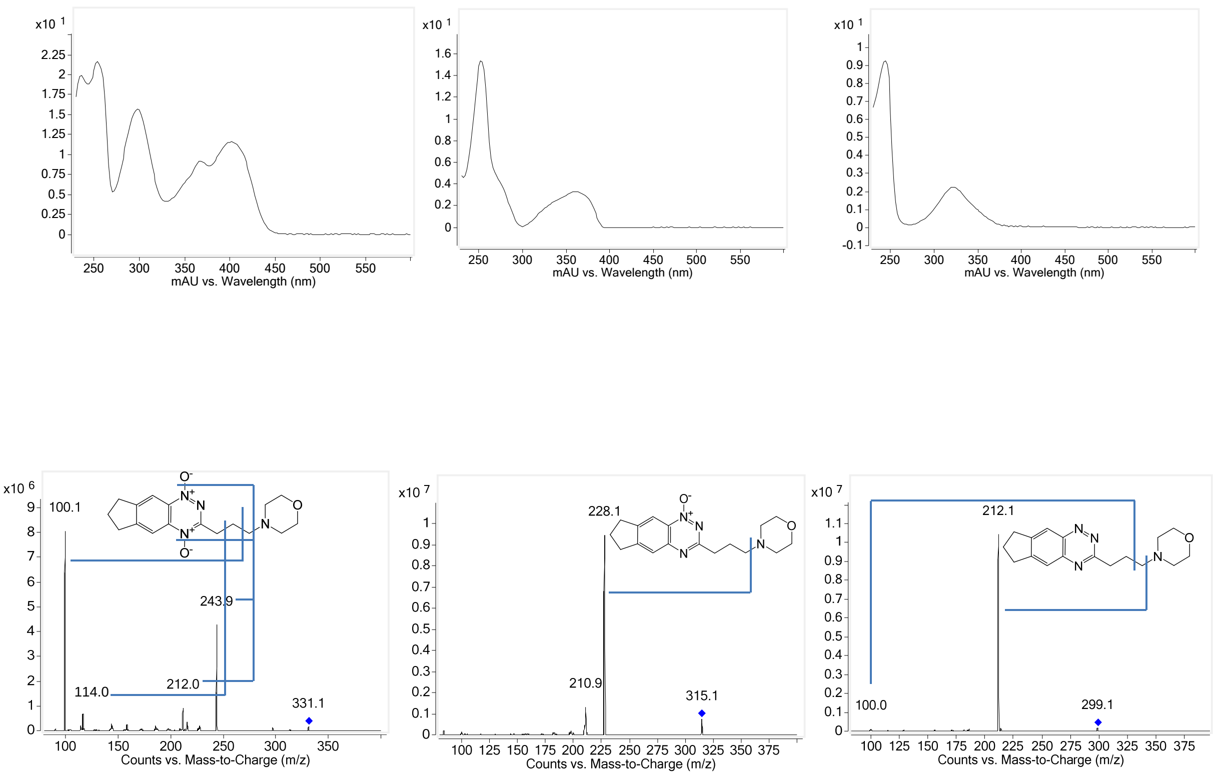

M1

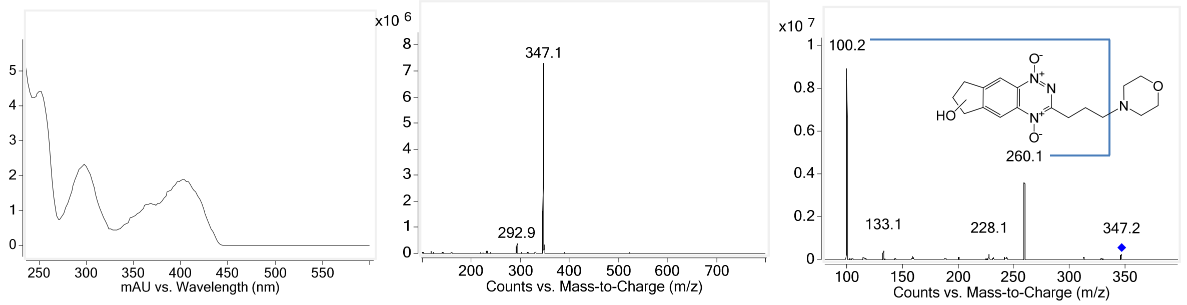

M2

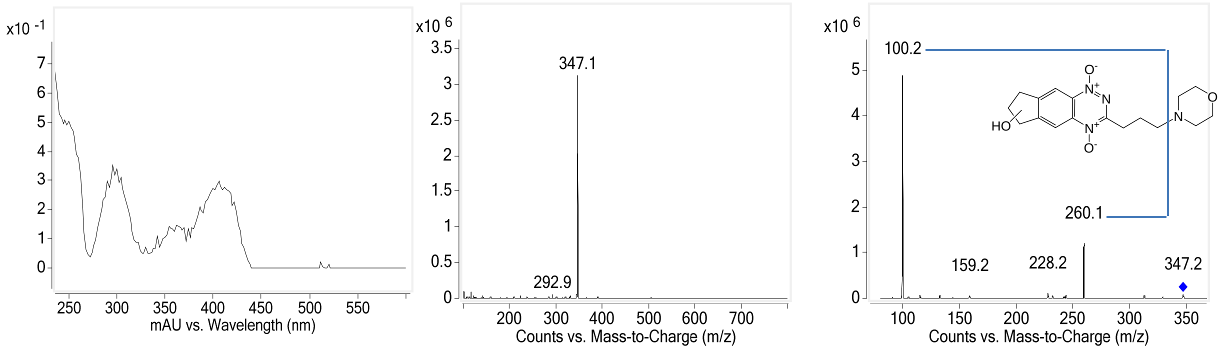
x10 -1
mAU vs. Wavelength (nm)
x10 6
347.1
292.9
Counts vs. Mass-to-Charge (m/z)
x10 6
100.2
260.1
159.2
228.2
347.2
HO
Counts vs. Mass-to-Charge (m/z)

M3

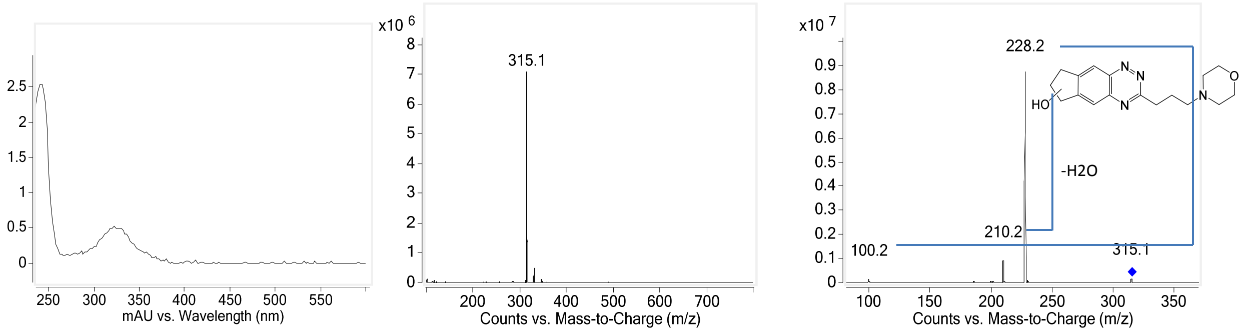
mAU vs. Wavelength (nm)
x10 6
315.1
Counts vs. Mass-to-Charge (m/z)
x10 7
228.2
HO
-H2O
210.2
100.2
315.1
Counts vs. Mass-to-Charge (m/z)

M4

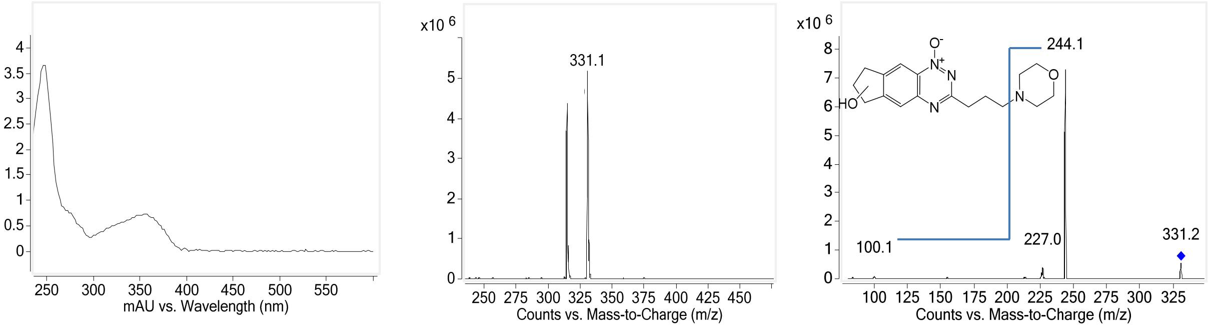
mAU vs. Wavelength (nm)
x10 6
331.1
Counts vs. Mass-to-Charge (m/z)
x10 6
244.1
HO
100.1
227.0
331.2
Counts vs. Mass-to-Charge (m/z)

M5

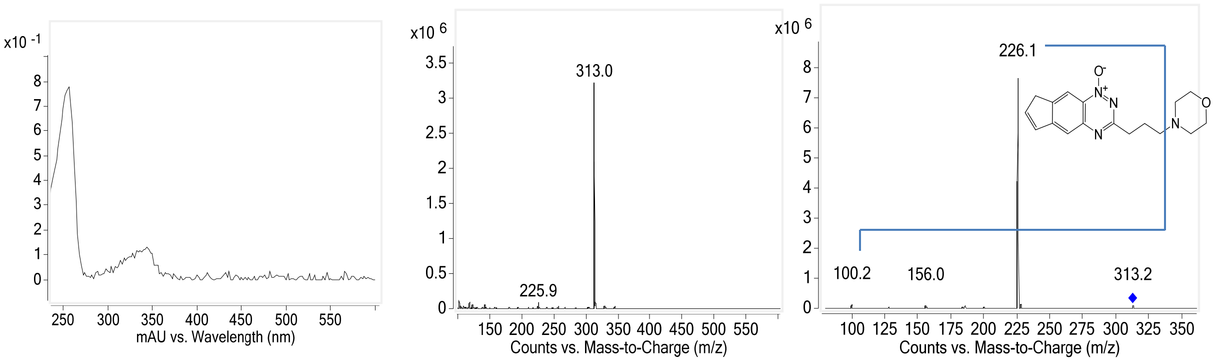
x10 -1
mAU vs. Wavelength (nm)
x10 6
313.0
225.9
Counts vs. Mass-to-Charge (m/z)
x10 6
226.1
100.2
156.0
313.2
Counts vs. Mass-to-Charge (m/z)

M6

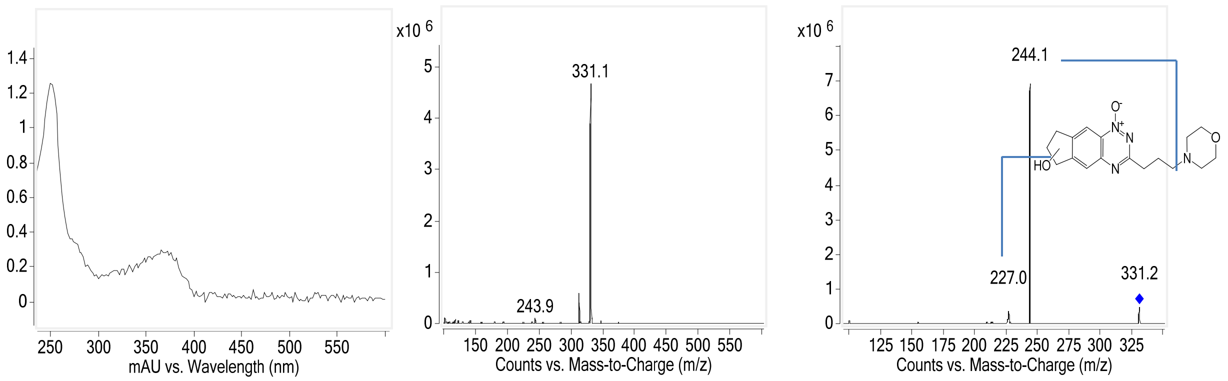
mAU vs. Wavelength (nm)
x10 6
331.1
243.9
Counts vs. Mass-to-Charge (m/z)
x10 6
244.1
HO
227.0
331.2
Counts vs. Mass-to-Charge (m/z)

M7

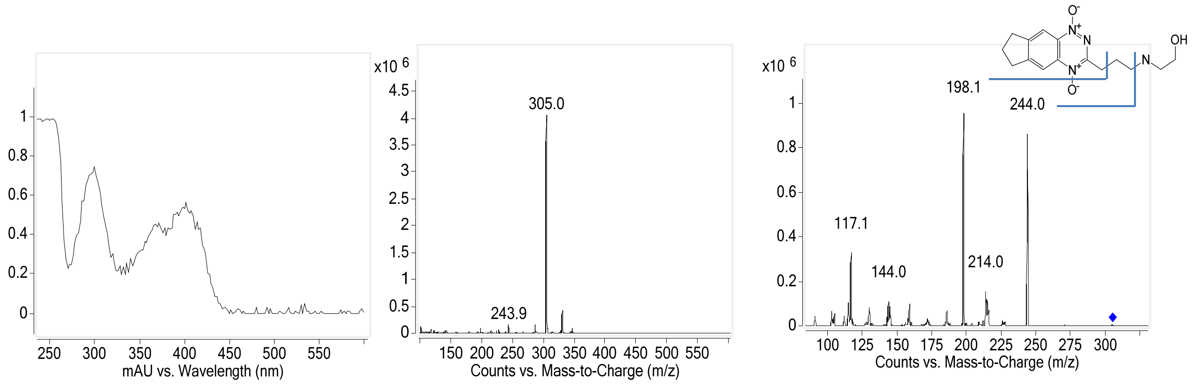
mAU vs. Wavelength (nm)
x10 6
305.0
243.9
Counts vs. Mass-to-Charge (m/z)
OH
x10 6
198.1
244.0
117.1
144.0
214.0
Counts vs. Mass-to-Charge (m/z)

M8

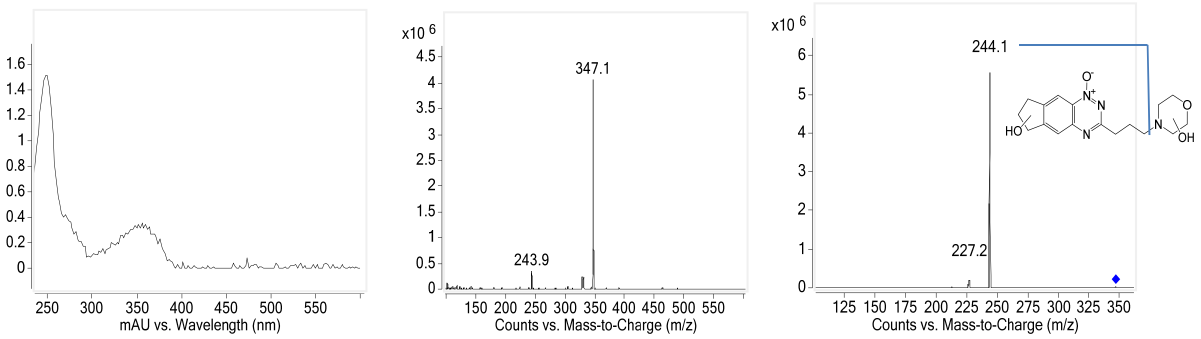
mAU vs. Wavelength (nm)
x10 6
347.1
243.9
Counts vs. Mass-to-Charge (m/z)
244.1
227.2
O-
N+
N
N
N
HO
O
OH
Counts vs. Mass-to-Charge (m/z)

M9

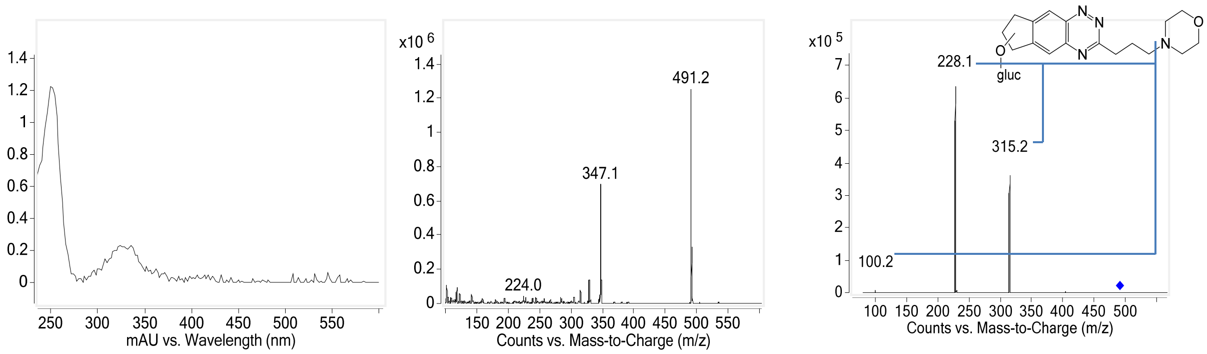
mAU vs. Wavelength (nm)
x10 6
491.2
347.1
224.0
Counts vs. Mass-to-Charge (m/z)
x10 5
228.1
315.2
100.2
O
gluc
N
O
Counts vs. Mass-to-Charge (m/z)

M10

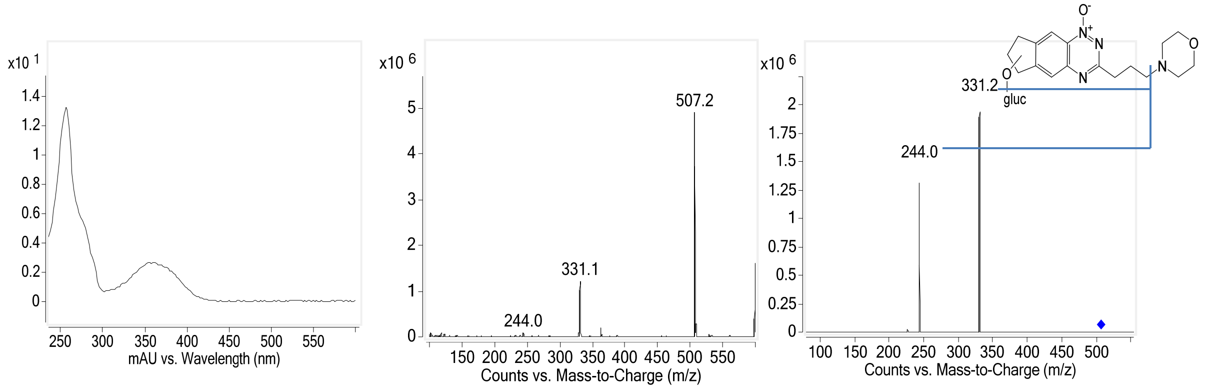
x10 1
mAU vs. Wavelength (nm)
x10 6
507.2
331.1
244.0
Counts vs. Mass-to-Charge (m/z)
x10 6
331.2
244.0
O-
N+
O
gluc
N
O
Counts vs. Mass-to-Charge (m/z)

M11

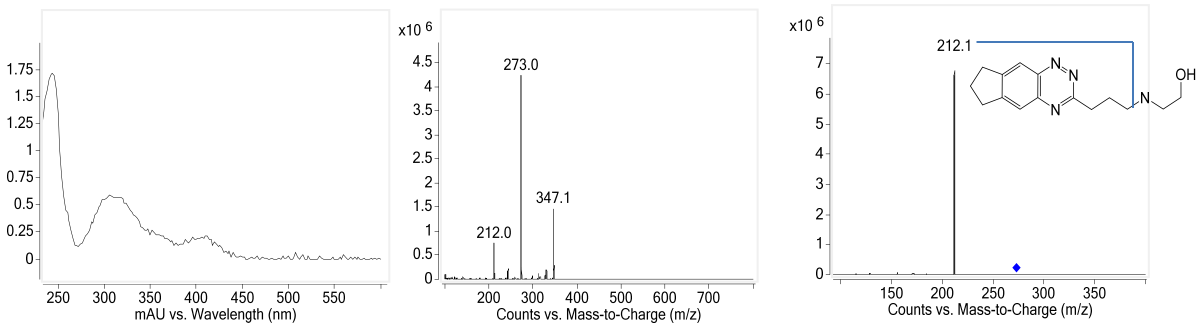
mAU vs. Wavelength (nm)
x10 6
273.0
347.1
212.0
Counts vs. Mass-to-Charge (m/z)
x10 6
212.1
N
N
N
N
OH
Counts vs. Mass-to-Charge (m/z)

M12

0

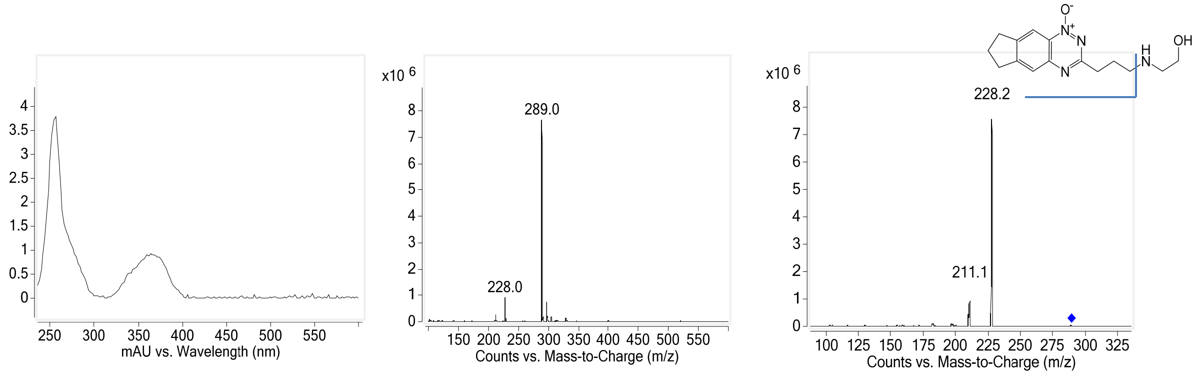
mAU vs. Wavelength (nm)
x10 6
289.0
228.0
Counts vs. Mass-to-Charge (m/z)
x10 6
228.2
211.1
O-
N+
N
N
H
N
OH
Counts vs. Mass-to-Charge (m/z)

M15

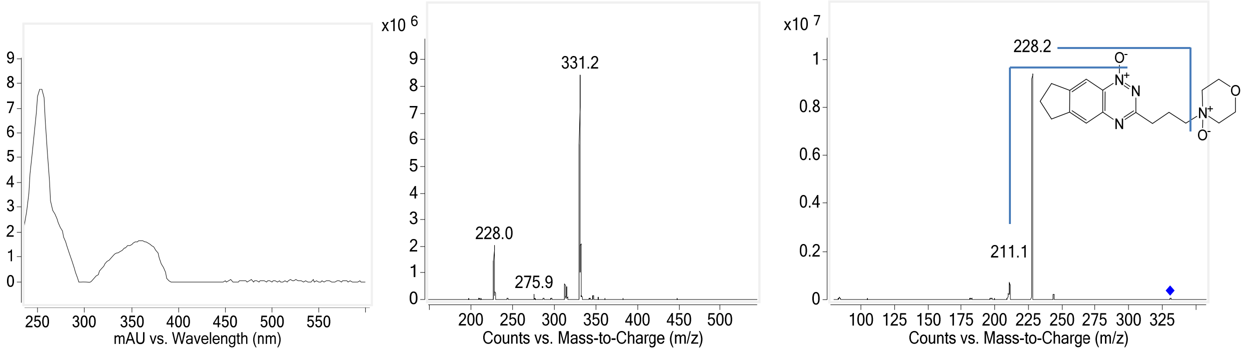
mAU vs. Wavelength (nm)
x10 6
331.2
228.0
275.9
Counts vs. Mass-to-Charge (m/z)
x10 7
228.2
211.1
O-
N+
N
N
N+
O-
O
Counts vs. Mass-to-Charge (m/z)

M16

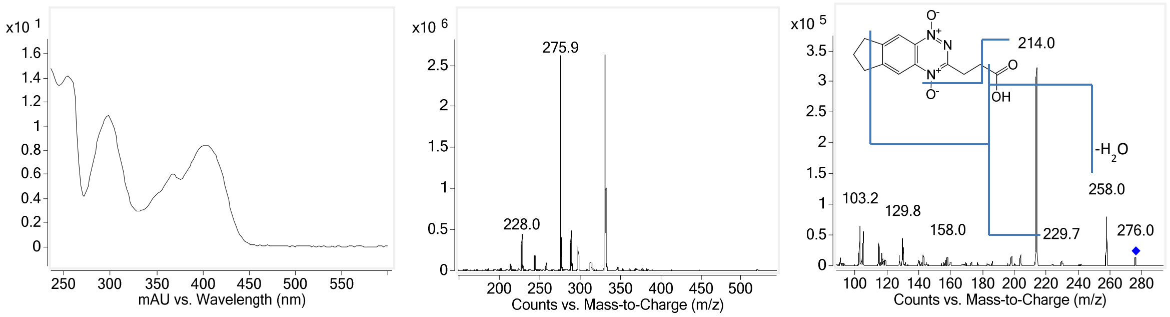
x10 1
mAU vs. Wavelength (nm)
x10 6
275.9
228.0
Counts vs. Mass-to-Charge (m/z)
x10 5
214.0
-H2O
258.0
103.2
129.8
158.0
229.7
276.0
OH
Counts vs. Mass-to-Charge (m/z)

M17

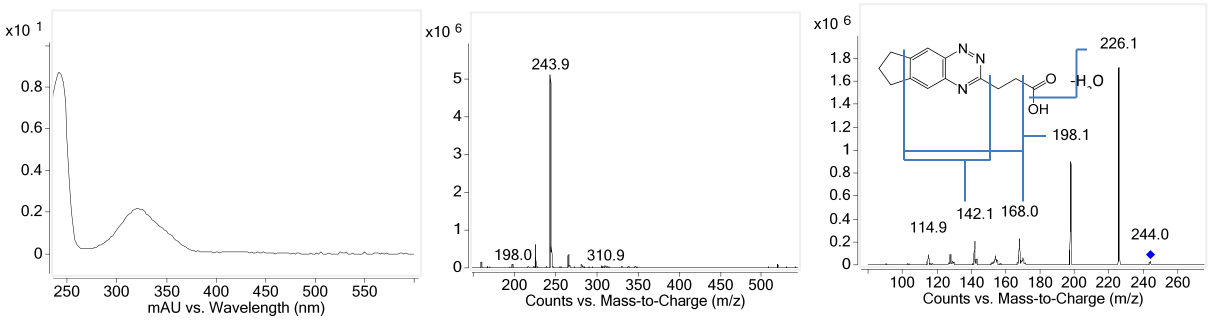
x10 1
mAU vs. Wavelength (nm)
x10 6
243.9
198.0
310.9
Counts vs. Mass-to-Charge (m/z)
x10 6
226.1
-H2O
OH
198.1
142.1
168.0
114.9
244.0
Counts vs. Mass-to-Charge (m/z)

M18

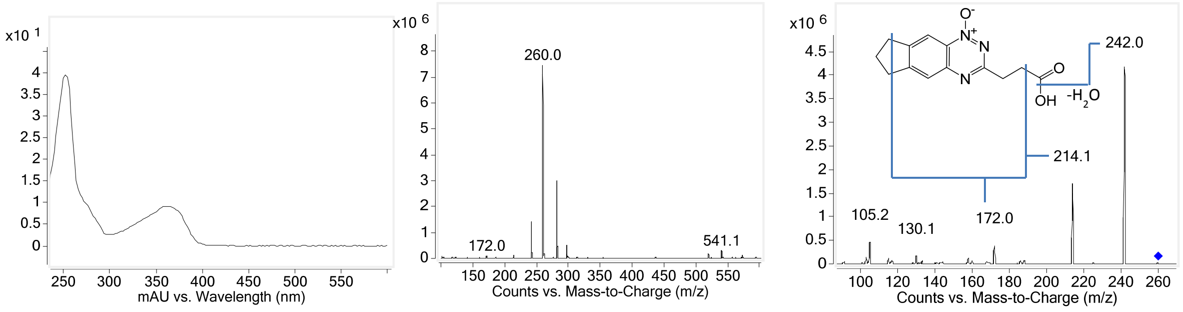
x10 1
mAU vs. Wavelength (nm)
x10 6
260.0
172.0
541.1
Counts vs. Mass-to-Charge (m/z)
x10 6
242.0
OH
-H2O
214.1
105.2
130.1
172.0
Counts vs. Mass-to-Charge (m/z)

M19

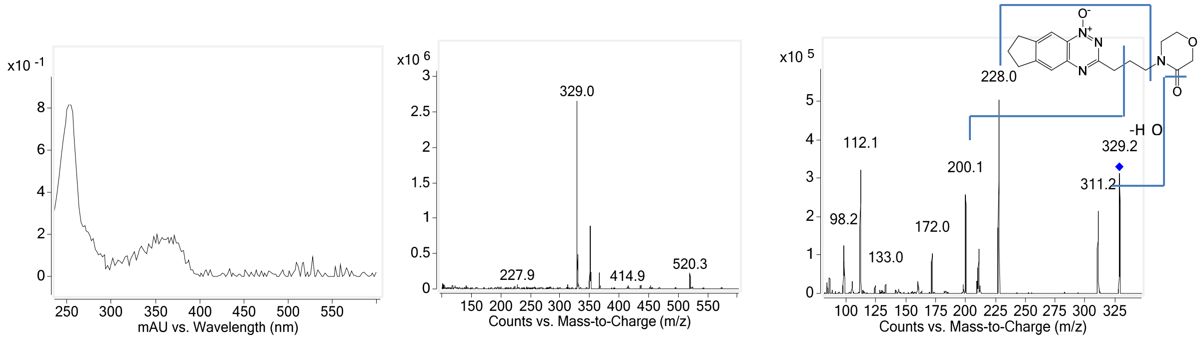
x10 -1
mAU vs. Wavelength (nm)
x10 6
329.0
227.9
414.9
520.3
Counts vs. Mass-to-Charge (m/z)
x10 5
228.0
112.1
200.1
-H O
329.2
311.2
98.2
172.0
133.0
Counts vs. Mass-to-Charge (m/z)

Supplementary Figure 3. Initial rates of core temperature decrease in (untreated) NIH-III mice post-mortem: comparison with initial temperature change ante-mortem following treatment with benzotriazine oxides at their MTD. Temperature was measured in rectum and liver over 15 min. Initial rates of temperature loss (°C/min) were: post-mortem (rectum): 0.65; post mortem (liver): 0.56; SN30000 186 mg/kg (rectum): 0.52; TPZ 31.7 mg/kg (rectum) 0.33. The rectal temperature loss following dosing with M14 (135 mg/kg) was 0.6 °C/min (not shown for clarity). The drug-induced changes are redrawn from Fig. 4.

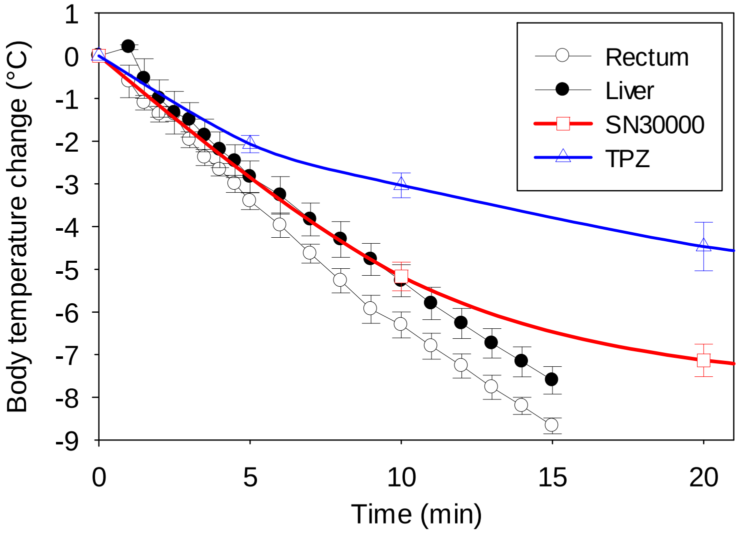

Supplementary Figure 4. Time-dependent changes in oxygen consumption and H_2O_2 formation rates induced by SN30000 in rotenone-inhibited CHO/POR cell suspensions, monitored with an OXYBOROS O2K oxygraph. Conditions of the experiment were identical to Fig. 5 except that SN30000 was added as a single bolus to separate cell suspension, rather than by cumulative addition. A: Representative traces for oxygen consumption and H_2O_2 formation rates for a single culture when SN30000 was added to a final concentration of 600 µM, at 4 min. Peak rates were established within 2 min, followed by a decline to a new steady state at approximately 1/3rd of the maximum rate. Cell viability was > 97% by trypan blue staining at the end of the SN30000 exposure, indicating that the fall was not due to cell killing. A potential mechanism of the time-dependent change is rapid depletion of NADPH pools through flavoreductase-dependent SN30000 reduction, with the slower steady state rate limited by the kinetics of regeneration of NADPH. B, C: Peak and steady state rates of non-respiratory (rotenone-insensitive) oxygen consumption (B) and H_2O_2 production (C) as a function of single bolus SN30000 concentrations. Dashed plots are redrawn from Fig. 5 for comparison with the response for cumulative addition of SN30000. Values are mean and SEM for 4 independent cell suspensions.

A:

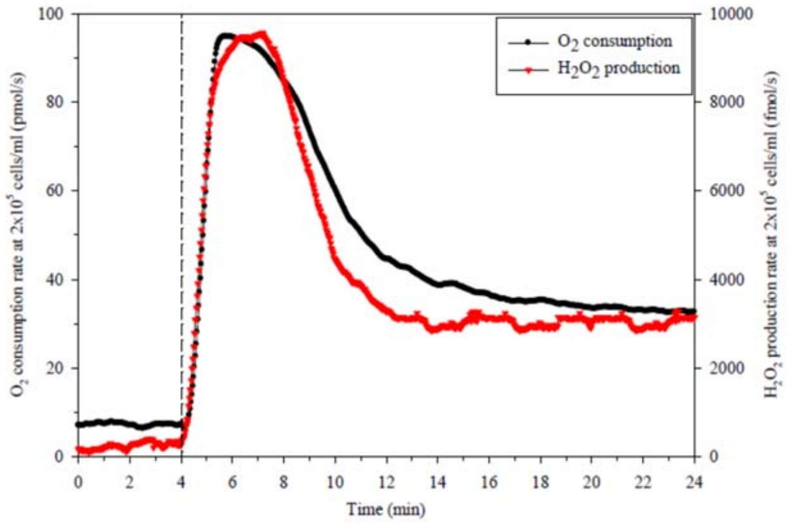

B:

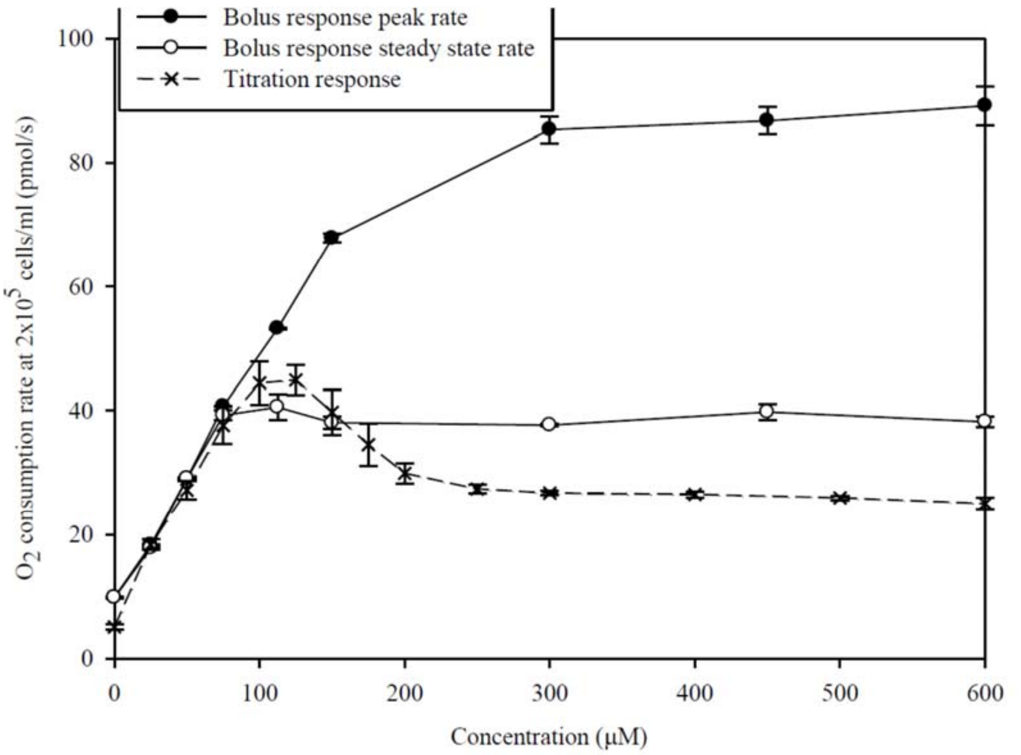
Bolus response peak rate
Bolus response steady state rate
Titration response
O2 consumption rate at 2x10^5 cells/ml (pmol/s)
0
20
40
60
80
100
0
100
200
300
400
500
600
Concentration (µM)

C:

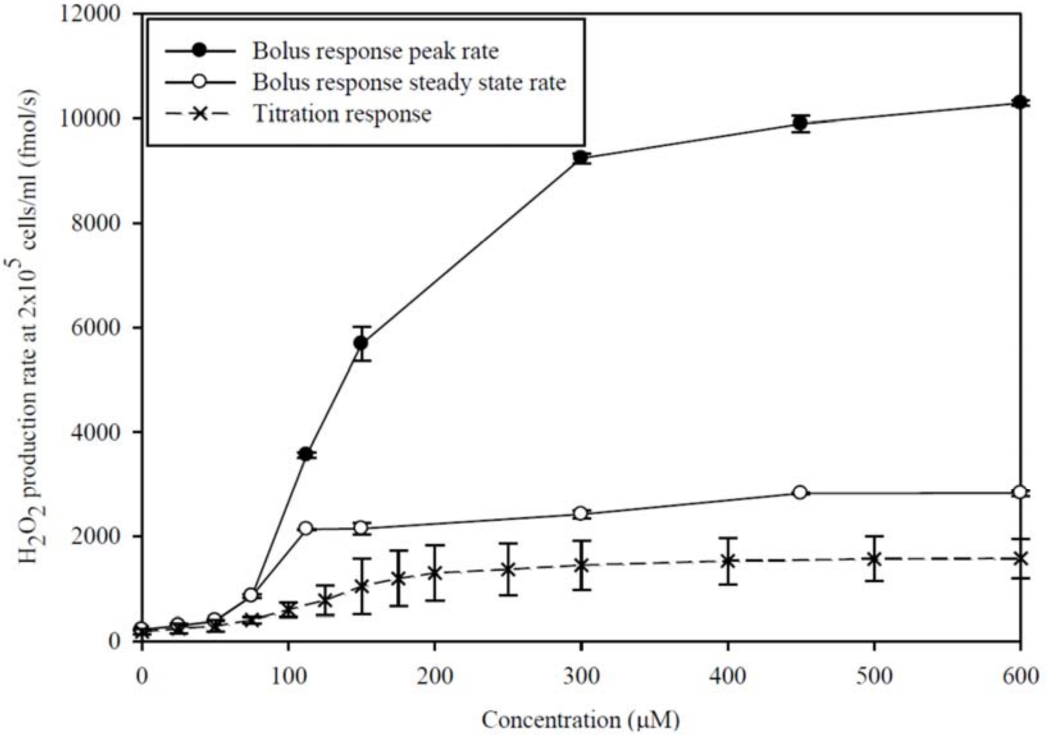
Bolus response peak rate
Bolus response steady state rate
Titration response
H2O2 production rate at 2x10^5 cells/ml (fmol/s)
0
2000
4000
6000
8000
10000
12000
0
100
200
300
400
500
600
Concentration (µM)

Supplementary Figure 5. Effects of SN30000, M14 and TPZ on oxygen consumption rates (OCR) of the human cervical carcinoma cell line SiHa and a previously described (Guise et al., 2007) POR-overexpressing transfectant SiHa/POR, determined using a Seahorse XFe96 Analyser (Agilent). Cells were passaged in αMEM plus 5% FCS, with 1 µM puromycin to maintain POR expression in the SiHa/POR cell line. Cells were typically seeded at 4×10^4 cells/well in Seahorse 96 well plates (without puromycin) and ~ 18 hr later the medium was changed to Seahorse medium with 10 mM glucose and 2 mM glutamine (SMGG) adjusted to pH 7.35 (150 µl/well). Plates were degassed to remove CO_2, and inhibitors were added serially using the Seahorse injectors (25 µl/injection) with rotenone (final concentration 0.5 µM) typically added at the start of the 30 min equilibration in the flux analyser. OCR was monitored for three 3 min periods following each drug addition.

A: Mitochondrial stress test for SiHa cells with SN30000, M14 (SN30672), or TPZ added at injection 1 and at each subsequent injection to maintain concentrations at 200 µM continuously during the test. The control received equivalent injections of SMGG without drugs. In all cases (including controls), injection 2 added oligomycin to give 0.5 µM, injection 3 added carbonyl cyanide 4-phenylhydrazone (FCCP) to 0.5 µM, and injection 4 added rotenone and antimycin to 1 µM each. Values are means and errors are standard deviations, for 6 replicate cultures. OCR was increased by SN30000 and TPZ, but suppressed by M14, both in the presence (injection 1) and absence (injection 4) of respiratory oxygen consumption.

B: Representative data demonstrating time-dependent changes in OCR by SiHa/POR cells in presence or absence of 0.5 µM rotenone when SN30000 is added cumulatively to give the indicated concentrations. Values are means and SEM for 5 replicate cultures. The fall in OCR after transient stimulation at each addition of SN30000 is consistent with the pattern for CHO/POR cells in Supplementary Fig. 4A.

C: Concentration dependence of increases in OCR by SiHa/POR cells, measured at the first time point after single injections of SN30000 or TPZ in order to minimise the time-dependent effects noted above. Left panel: SN30000. Right panel: TPZ. Values are means and errors are SEM for 3-5 cultures.

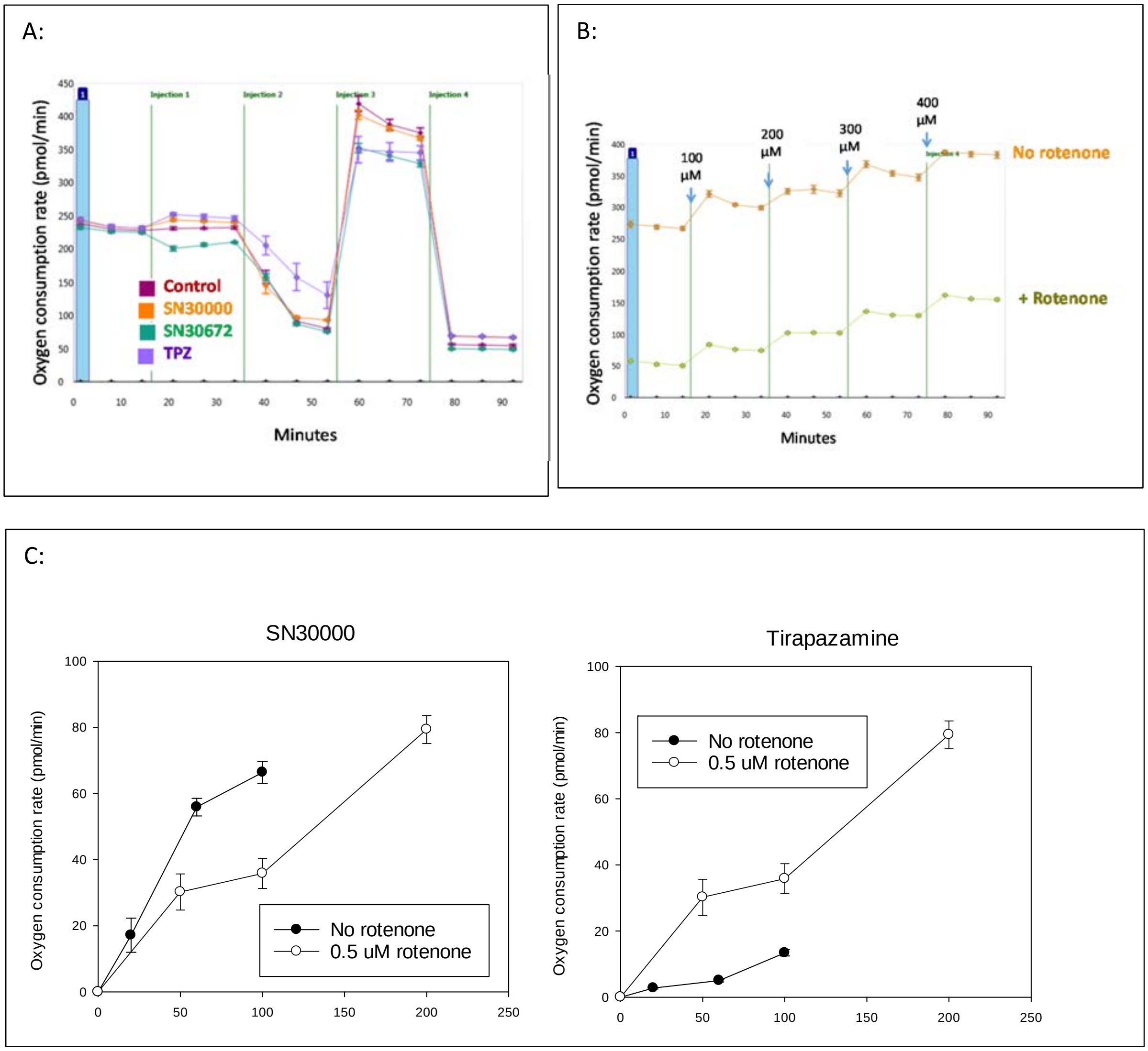
A:
Injection 1
Injection 2
Injection 3
Injection 4
Oxygen consumption rate (pmol/min)
Control
SN30000
SN30672
TPZ
Minutes
B:
100 µM
200 µM
300 µM
400 µM
No rotenone
+ Rotenone
Oxygen consumption rate (pmol/min)
Minutes
C:
SN30000
Tirapazamine
Oxygen consumption rate (pmol/min)
No rotenone
0.5 uM rotenone

Supplementary Figure 6. Post-mortem metabolism of SN30000 and M14 in female NIH-III mice: time dependence of tissue concentrations. Concentrations of SN30000 and its major reduced metabolites in liver (A) and HT29 tumour (B) tissue sampled after i.p. administration of SN30000 at 186 mg/kg, or in liver (C) after i.p. administration of M14 at 135 mg /kg. Mice were culled 30 min (SN30000) or 60 min (M14) after dosing and held at ambient temperature for the indicated times before sampling. Results are expressed as percentages of the respective concentrations when sampling was immediate. Values are means and SEM for ≥3 animals. * and *** denote $P < 0.05$ and <0.001 respectively with respect to the initial values using 1-way ANOVA for each compound with the Holm-Sidak multiple comparison test.

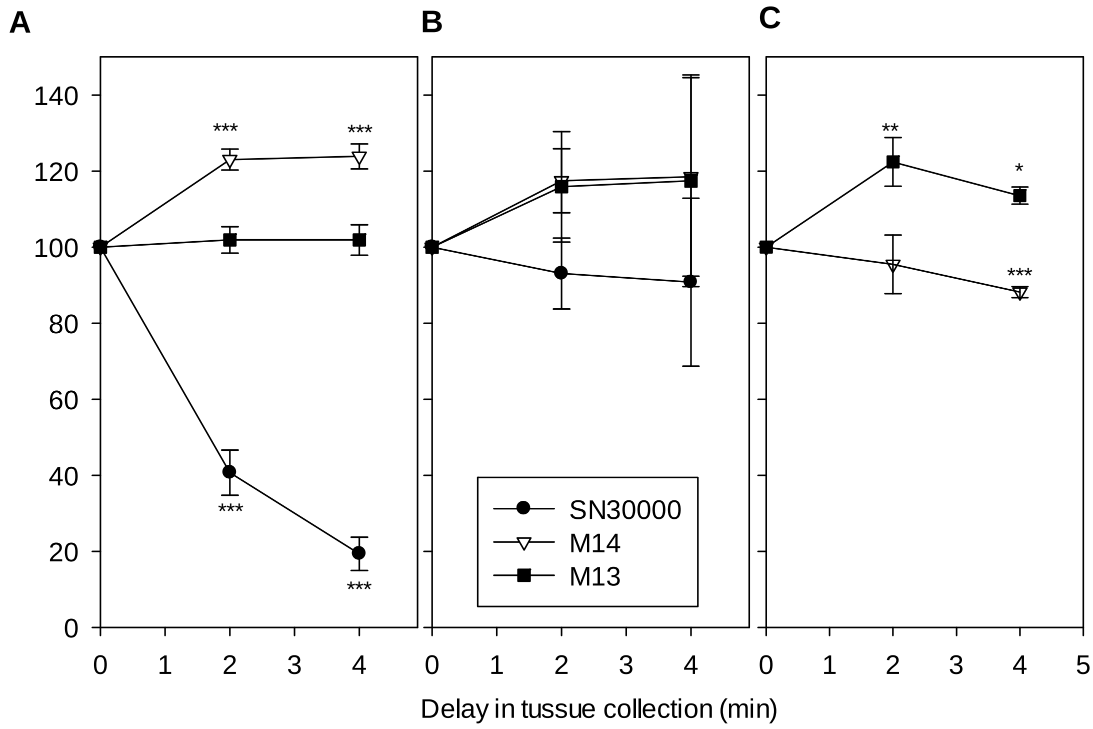

Supplementary Figure 7. Post-mortem metabolism of SN30000 in female NIH-III mice: dependence on sequence of tissue sampling. Mice were treated with SN30000 at 186 mg/kg by i.p. administration 30 min before termination. A: SN30000, 1-oxide M14 and nor-oxide M13 concentrations in liver. B: M14/SN30000 ratio in plasma, liver and brain tissues. Group 1: Sampling of liver, blood and brain (in that sequence) under ketamine/xylazine anaesthesia. Group 2: Sampling of brain, blood and liver (in that sequence) under ketamine/xylazine anaesthesia. Group 3: Cervical dislocation followed by sampling of blood, liver and brain. Mice were held at ambient temperature after culling. Values are means and errors are SEM derived from 4 animals per group. * and *** denote $P < 0.05$ and 0.001 respectively. Post-mortem metabolism was significant only in liver, and was least when liver was sampled first.

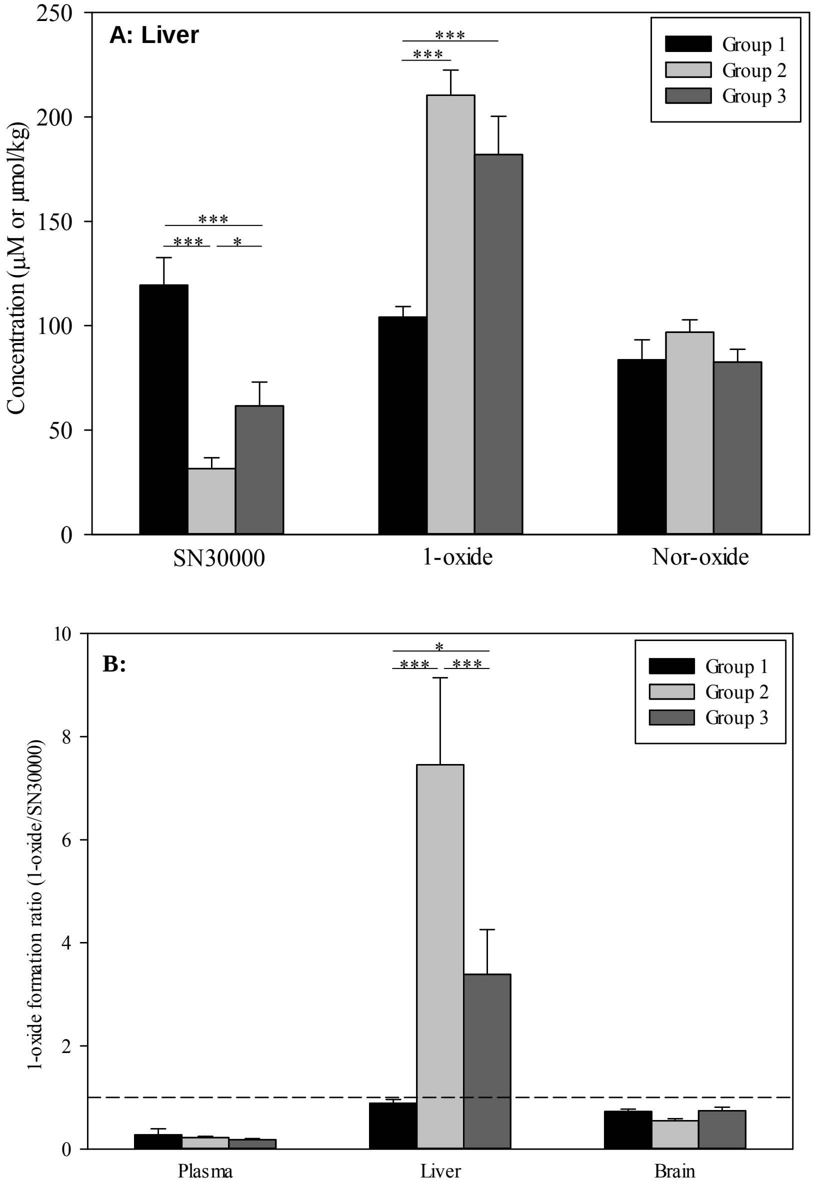

Supplementary Figure 8. Concentration-time profiles in plasma for SN30000 (A) and metabolites M14 (B), M13 (C) and M18 (D) after i.v. dosing of female NIH-III mice with SN30000. Values are mean ± SEM for 3 mice at each time. Lines are fitted using the compartmental model (Fig. 9) with the parameters shown in Supplementary Table 12.

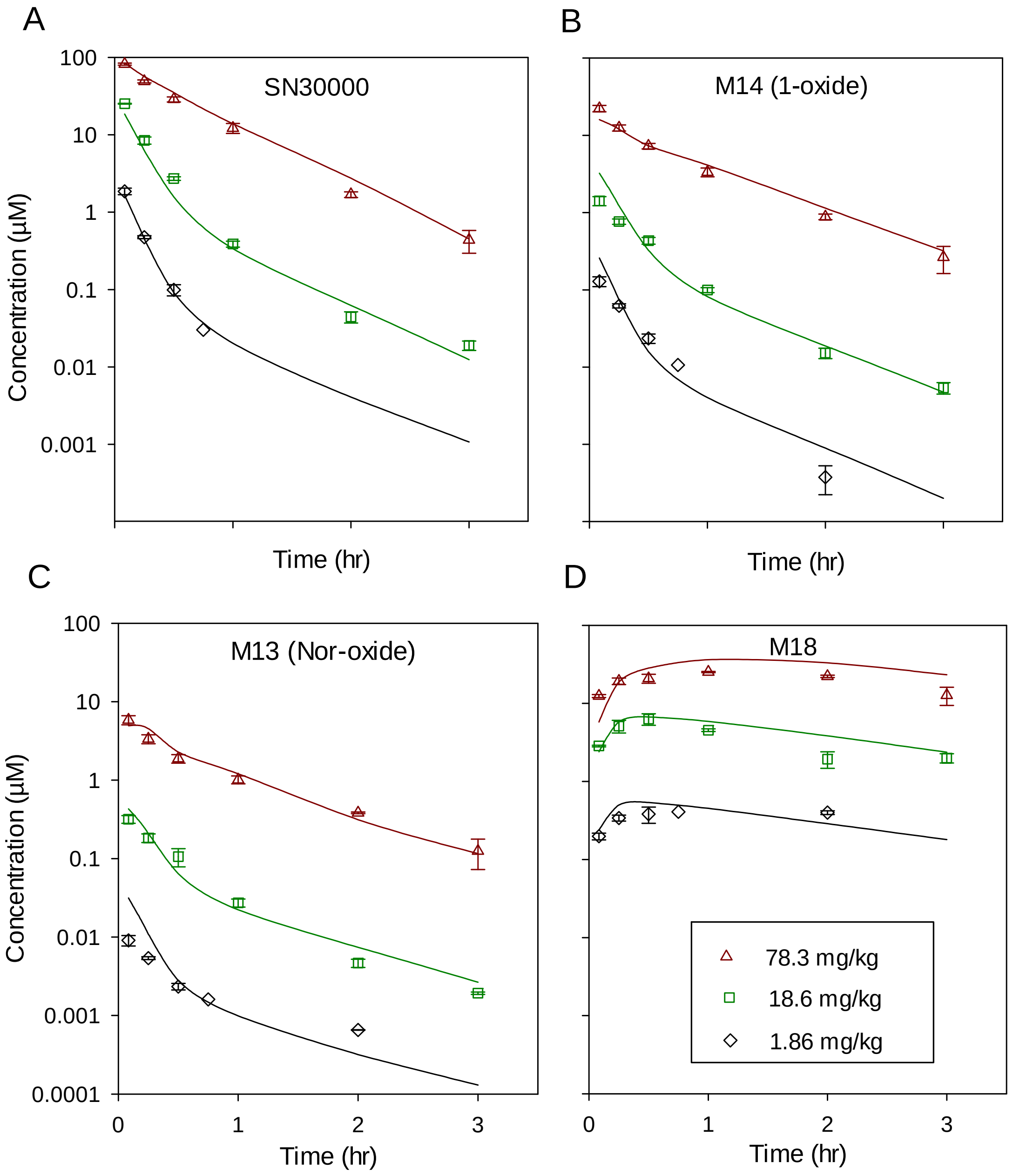

Supplementary Figure 9. Plasma concentration-time profiles for M14, dosed at 135 mg/kg i.p., redrawn from Fig.3 with curves fitted using the temperature-dependent compartmental model (Fig. 9). Model parameters are listed in Supplementary Table 12.

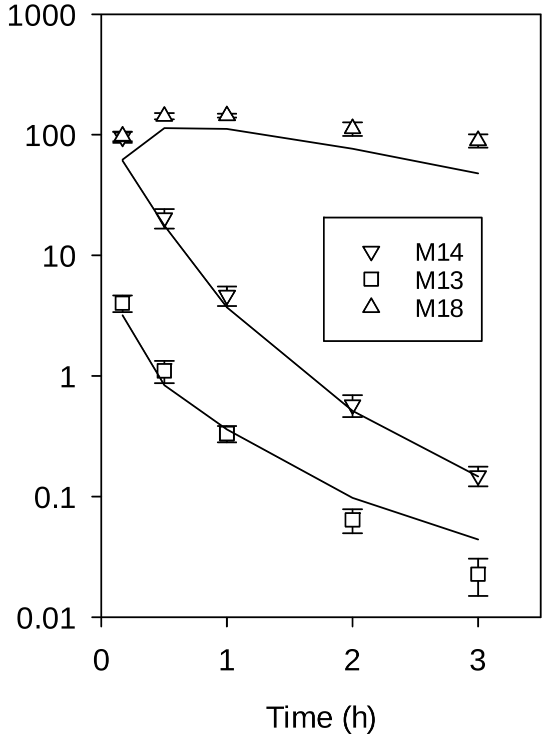

SUPPLEMENTARY REFERENCES

Banker, M.J., Clark, T.H., and Williams, J.A. (2003). Development and validation of a 96-well equilibrium dialysis apparatus for measuring plasma protein binding. *J. Pharm. Sci.* 92, 967-974.

Gu, Y., Jaiswal, J.K., Wang, J., Hicks, K.O., Hay, M.P. and Wilson, W.R. (2014). Photodegradation of the benzotriazine 1,4-di-N-oxide hypoxia-activated prodrug SN30000 in aqueous solution. *J. Pharm. Sci.* 103, 3464-3472.

Gu, Y., Patterson, A.V., Atwell, G.J., Chernikova, S.B., Brown, J.M., Thompson, L.H., et al. (2009). Roles of DNA repair and reductase activity in the cytotoxicity of the hypoxia-activated dinitrobenzamide mustard PR-104A. *Mol. Cancer Ther.* 8, 1714-1723.

Guise, C.P., Wang, A., Thiel, A., Bridewell, D., Wilson, W.R., and Patterson, A.V. (2007). Identification of human reductases that activate the dinitrobenzamide mustard prodrug PR-104A: a role for NADPH:cytochrome P450 oxidoreductase under hypoxia. *Biochem. Pharmacol.* 74, 810-820.

Hay, M.P., Hicks, K.O., Pchalek, K., Lee, H.H., Blaser, A., Pruijn, F.B., et al. (2008). Tricyclic 1,2,4-triazine 1,4-dioxides as hypoxia selective cytotoxins. *J. Med. Chem.* 51, 6853–6865.

Obregon, A.D.C., Schetinger, M.R.C., Correa, M.M., Morsch, V.M., da Silva, J.E.P., Martins, M.A.P., et al. (2005). *Neurochem. Res.* 30, 379–384.

Wang, J., Foehrenbacher, A., Su, J., Patel, R., Hay, M.P., Hicks, K.O., et al. (2012). The 2-nitroimidazole EF5 is a biomarker for oxidoreductases that activate bioreductive prodrug CEN-209 under hypoxia. *Clin. Cancer Res.* 18, 1684-1695.
